# Guided cobamide biosynthesis for heterologous production of reductive dehalogenases

**DOI:** 10.1111/1751-7915.13339

**Published:** 2018-12-13

**Authors:** Torsten Schubert, Stephan H. von Reuß, Cindy Kunze, Christian Paetz, Stefan Kruse, Peggy Brand‐Schön, Anita Mac Nelly, Jörg Nüske, Gabriele Diekert

**Affiliations:** ^1^ Department of Applied and Ecological Microbiology Institute of Microbiology Friedrich Schiller University Philosophenweg 12 D‐07743 Jena Germany; ^2^ Department of Bioorganic Chemistry Max Planck Institute for Chemical Ecology Hans‐Knöll‐Straße 8 D‐07745 Jena Germany; ^3^ Research Group Biosynthesis/NMR Max Planck Institute for Chemical Ecology Hans‐Knöll‐Straße 8 D‐07745 Jena Germany; ^4^ Present address: Laboratory for Bioanalytical Chemistry Institute of Chemistry University of Neuchâtel Avenue de Bellevaux 51 2000 Neuchâtel Switzerland; ^5^ Present address: DECHEMA‐Forschungsinstitut Theodor‐Heuss‐Allee 25 D‐60486 Frankfurt am Main Germany

## Abstract

Cobamides (Cbas) are essential cofactors of reductive dehalogenases (RDases) in organohalide‐respiring bacteria (OHRB). Changes in the Cba structure can influence RDase function. Here, we report on the cofactor versatility or selectivity of *Desulfitobacterium* RDases produced either in the native organism or heterologously. The susceptibility of *Desulfitobacterium hafniense* strain DCB‐2 to guided Cba biosynthesis (*i.e*. incorporation of exogenous Cba lower ligand base precursors) was analysed. Exogenous benzimidazoles, azabenzimidazoles and 4,5‐dimethylimidazole were incorporated by the organism into Cbas. When the type of Cba changed, no effect on the turnover rate of the 3‐chloro‐4‐hydroxy‐phenylacetate‐converting enzyme RdhA6 and the 3,5‐dichlorophenol‐dehalogenating enzyme RdhA3 was observed. The impact of the amendment of Cba lower ligand precursors on RDase function was also investigated in *Shimwellia blattae*, the Cba producer used for the heterologous production of *Desulfitobacterium* RDases. The recombinant tetrachloroethene RDase (PceA_Y51_) appeared to be non‐selective towards different Cbas. However, the functional production of the 1,2‐dichloroethane‐dihaloeliminating enzyme (DcaA) of *Desulfitobacterium dichloroeliminans* was completely prevented in cells producing 5,6‐dimethylbenzimidazolyl‐Cba, but substantially enhanced in cells that incorporated 5‐methoxybenzimidazole into the Cba cofactor. The results of the study indicate the utilization of a range of different Cbas by *Desulfitobacterium* RDases with selected representatives apparently preferring distinct Cbas.

## Introduction

Reductive dehalogenase enzymes (RDases) in organohalide‐respiring bacteria (OHRB) (Hug *et al*., 2013) are environmentally relevant enzymes due to their role in the detoxification of halogenated organic pollutants in anoxic soil or groundwater (Jugder *et al*., 2016; Fincker and Spormann, 2017; Schubert *et al*., 2018; Wang *et al*., 2018). OHRB were successfully applied in bioremediation of contaminated field sites (Major *et al*., 2002; Lendvay *et al*., 2003; Löffler and Edwards, 2006). Hence, the understanding of the structure–function relationship of RDases is of substantial interest. The biochemical characterization of RDase enzymes (Jugder *et al*., 2016; Fincker and Spormann, 2017; Schubert *et al*., 2018; Wang *et al*., 2018) and their biotechnological application (Siritanaratkul *et al*., 2016) is often hampered by the intricate handling of the oxygen‐sensitive enzymes and the limited amount of biomass produced by OHRB when cultivated with organohalides.

RDases are iron–sulfur proteins, which bind a cobamide (Cba; B_12_ vitamer) cofactor in a structurally conserved nitroreductase fold (Bommer *et al*., 2014; Payne *et al*., 2015). Since the first RDase‐encoding gene was identified (Neumann *et al*., 1998), heterologous production of catalytically active RDases was attempted, in order to simplify RDase purification. However, these early experiments were unsuccessful, most probably due to the use of *Escherichia coli* as the expression host (Neumann *et al*., 1998; Suyama *et al*., 2002; Kimoto *et al*., 2010; Sjuts *et al*., 2012; Parthasarathy *et al*., 2015). *Escherichia coli* is not able to synthesize Cbas *de novo* (Blattner *et al*., 1997). Hence, a host organism for heterologous production had to be found that provided both an adequate amount of Cbas and iron–sulfur clusters for RDase assembly. In recent years, the Cba‐producing gammaproteobacterium *Shimwellia blattae* (Lawrence and Roth, 1996) and the Gram‐positive *Bacillus megaterium* (Wolf and Brey, 1986) were successfully applied (Mac Nelly *et al*., 2014; Payne *et al*., 2015; Kunze *et al*., 2017; Jugder *et al*., 2018). *B. megaterium* synthesizes the standard‐type B_12_ cofactor (5,6‐dimethylbenzimidazolyl‐Cba), and *S. blattae* produces pseudo‐B_12_ (adeninyl‐Cba) *de novo* (Wolf and Brey, 1986; Mac Nelly *et al*., 2014).

Natural Cbas are ‘complete’ corrinoids (structural variants of vitamin B_12_) that consist of a contracted tetrapyrrole ring system tethered to a nucleotide loop (Lenhert and Hodgkin, 1961). Structural diversity among natural Cbas is mainly based on the incorporation of various bases such as benzimidazoles (Bzas), purines or phenolic compounds into the nucleotide loop substructure (Renz, 1999). Cba structure can be modulated by the so‐called ‘guided biosynthesis’, *i.e*. feeding building blocks such as lower base precursors (mostly Bzas) to the growing cells of a given Cba producer (Pawelkiewicz and Nowakowska, 1955; Perlman and Barrett, 1958; Stupperich *et al*. 1987; Mok and Taga, 2013; Keller *et al*., 2014, 2018). Changes in the Cba structure can interfere with RDase function in OHRB (Yan *et al*., 2012, 2013, 2016; Yi *et al*., 2012; Keller *et al*., 2014) most probably due to an incompatibility of the cofactor with a correct enzyme folding (Keller *et al*., 2018).

In general, Cba‐containing enzymes bind the cofactor in two different modes: the base‐on conformation with the lower base (Bzas or purines) of the nucleotide loop establishing a coordinative bond with the central cobalt ion (eliminases, ribonucleotide reductase) or the base‐off conformation with the lower base displaced from the cobalt (methyl transferases, reductive dehalogenases, epoxyqueuosine reductase) (Banerjee and Ragsdale, 2003; Gruber *et al*., 2003; Bridwell‐Rabb and Drennan, 2017). Structural analysis of the tetrachloroethene RDase (PceA) from the epsilonproteobacterium *Sulfurospirillum multivorans* (Bommer *et al*., 2014) and the bromophenol RDase from the marine alphaproteobacterium *Nitratireductor pacificus* (NpRdhA) (Payne *et al*., 2015) uncovered the Cba cofactor bound in the base‐off state with the nucleotide loop involved in enzyme‐cofactor binding rather than in the catalytic cycle.

Among the OHRB, Cba producers and Cba auxotrophs were identified. Cba production has been proven for *S*. *multivorans* (Kräutler *et al*., 2003; Keller *et al*., 2014) and *S. halorespirans* (Goris *et al*., 2017), *D. hafniense* strains Y51, JH1, Viet1 and PCE1 (Reinhold *et al*., 2012; Yan *et al*. 2018), *Geobacter lovleyi* (Yan *et al*., 2012) and *Dehalobacter* sp. strains TCA1, CF, DCA and UNSWDHB (Sun *et al*., 2002; Tang *et al*., 2016; Wong *et al*., 2016; Wang *et al*., 2017). The organohalide‐respiring *Dehalobacter restrictus* (Rupakula *et al*., 2013) and *Dehalococcoides mccartyi* (Löffler *et al*., 2013) are Cba auxotrophs that strictly depend on the uptake of Cbas from the environment.

Besides genes encoding RDases, the reductively dehalogenating representatives of the genus *Desulfitobacterium* possess the genetic information needed for Cba biosynthesis (Kruse *et al*., 2017). Genes for the biosynthesis of Bzas were not identified, which is consistent with the finding that selected *D. hafniense* strains produce a Cba with unsubstituted purine as lower base (Yan *et al*., 2018). To date, three RDases from *Desulfitobacterium* species were heterologously produced in a catalytically active state in *S. blattae*: the tetrachloroethene RDase of *D. hafniense* strain Y51 (PceA_Y51_), the 3,5‐dichlorophenol RDase of *D. hafniense* strain DCB‐2 (RdhA3) and the 1,2‐dichloroethane RDase of *Desulfitobacterium dichloroeliminans* (DcaA) (Mac Nelly *et al*., 2014; Kunze *et al*., 2017). Up to date, little is known about the versatility or specificity in cofactor utilization by *Desulfitobacterium* RDases, an information that might be a key to their functional heterologous production. In the study presented here, *D. hafniense* strain DCB‐2 and the host for the expression of *Desulfitobacterium* RDases, namely *S. blattae*, were analysed for their susceptibility to guided Cba biosynthesis. The influence of cultivation conditions (*i.e*. medium ingredients) on Cba cofactor production in both organisms was investigated. Furthermore, the impact of different Cbas on RDase function was examined. As a result, among *Desulfitobacterium* RDases, which in three cases remained uninfluenced in the presence of different Cbas, a single representative was identified that showed a clear preference in Cba cofactor utilization. Overall, this study brings forth guided Cba biosynthesis as a biotechnological tool for the improvement of the heterologous production of RDases.

## Results

### Guided cobamide biosynthesis and reductive dehalogenase activity in *D. hafniense* strain DCB‐2

**Figure 1:**
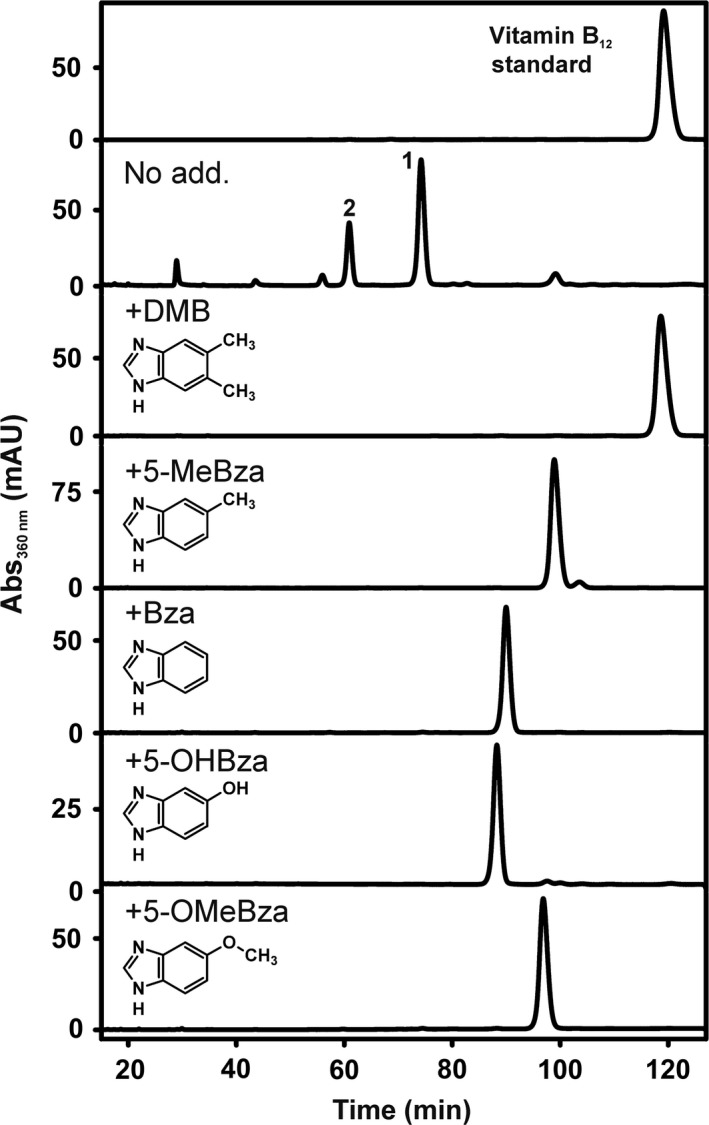
HPLC analysis of Cbas extracted from *D. hafniense* strain DCB‐2 grown with pyruvate and ClOHPA. DMB = 5,6‐dimethylbenzimidazole, 5‐MeBza = 5‐methylbenzimidazole, Bza = benzimidazole, 5‐OHBza = 5‐hydroxybenzimidazole, 5‐OMeBza = 5‐methoxybenzimidazole (25 μM respectively).

For the analysis of cobamide (Cba) biosynthesis, the type strain of the species *D. hafniense*, namely strain DCB‐2, was chosen and cultivated in the absence or presence of exogenous Cba lower ligand base precursors. Pyruvate (electron donor) and 3‐chloro‐4‐hydroxy‐phenylacetate (ClOHPA; electron acceptor) were applied as growth substrates, and 0.2% yeast extract (YE) was added to the cultures. The presence of YE is not essential for the growth of *D. hafniense* strain DCB‐2 with pyruvate as electron donor, but it substantially increases the growth yield (Madsen and Licht, 1992). The Cbas, which were produced by the organism, were extracted from whole cells in the presence of cyanide, purified and analysed via high‐performance liquid chromatography (HPLC) (Fig. 1), UV/Vis spectroscopy (Fig. S1) and electrospray ionization–high‐resolution tandem mass spectrometry (ESI‐HR‐MS/MS) (Fig. S2 and Tables S1–S8). Two Cbas (**1** and **2** in Fig. 1) were identified in cells cultivated in the absence of Cba lower ligand base precursors. While MS/MS analysis of **1** pointed towards purine (C_5_H_5_N_4_) as lower base (Table S2), the incorporation of an azabenzimidazole (azaBza) (C_6_H_6_N_3_) was indicated for **2** (Table S3). Analysis of one‐ and two‐dimensional nuclear magnetic resonance (NMR) spectra of the purified compounds allowed for the unambiguous identification of the Cba structures (Table S13). Comparative analysis of the double‐quantum filtered correlation spectroscopy (dqf‐COSY) spectra with those of a vitamin B_12_ standard confirmed the identification of the Cba skeleton. The structure of the lower ligands was deduced from the heteronuclear multiple bond correlation (HMBC) spectra (Figs S3 and S4), which confirmed the identification of purinyl‐Cba (**1**) and 5‐azaBza‐Cba (**2**), whereas the three‐dimensional arrangement of the lower ligands was finally derived from nuclear Overhauser effect spectroscopy (NOESY) spectra (Fig. S5). This result confirmed the recent report on purinyl‐Cba production in *D. hafniense* strains Y51, JH1, Viet1 and PCE1 (Yan *et al*., 2018). The production of the 5‐azaBza‐Cba in *D. hafniense* strain DCB‐2 was a novel finding, although the incorporation of azabenzimidazoles as Cba lower ligands was reported earlier by Endres *et al*. (1995), who added 4‐azaBza to cultures of the non‐dehalogenating acetogen *Eubacterium limosum*.

In order to investigate guided Cba biosynthesis in *D. hafniense* strain DCB‐2, cells were cultivated in the presence of different Bzas. When the Cbas were extracted from such cells, an average yield of 0.40 ± 0.15 μmol Cba per g protein was obtained that was about one‐third of the amount previously reported for the norcobamide‐producing OHRB *Sulfurospirillum multivorans* (Keller *et al*., 2014). As shown in Figure 1, the presence of exogenous Bzas (25 μM each) in cultures grown on pyruvate, ClOHPA and YE resulted in the absence of the initially identified Cba mixture including purinyl‐Cba (**1**) and 5‐azaBza‐Cba (**2**) and led to the formation of alternative Cbas that harboured the Bza added to the growth medium as the lower base as verified by ESI‐HR‐MS/MS (Tables S4–S8). In order to test for the positioning of the singly substituted Bzas in the Cba structures, ^1^H‐NMR spectra were recorded (Fig. S6). The production of 5‐methylbenzimidazolyl (MeBza)‐Cba or 5‐methoxybenzimidazolyl (OMeBza)‐Cba was confirmed (Crofts *et al*., 2014a; Keller *et al*., 2018). In the Cba sample obtained from cells cultivated in the presence of 5‐hydroxybenzimidazole (OHBza), a mixture of both Cba isomers, the 5‐OHBza‐Cba (62.5%) and the 6‐OHBza‐Cba (37.5%) isomer, was detected, which could not be efficiently separated upon liquid chromatography (Fig. 1).

Five RDases are encoded in the *D. hafniense* strain DCB‐2 genome (Kim *et al*., 2012). In the presence of ClOHPA as electron acceptor, the *rdhA6* gene is transcribed (Fig. S6), which encodes the ClOHPA‐RDase (Christiansen *et al*., 1998), whereas in the presence of 3,5‐dichlorophenol (3,5‐DCP) exclusively *rdhA3* is expressed (Mac Nelly *et al*., 2014). RdhA3 is a chlorophenol‐converting RDase that dehalogenates 3,5‐DCP to 3‐chlorophenol. To unravel the effect of structurally different Cbas on the function of both RDases, the enzyme activity was tested in crude extracts obtained from cells cultivated with either ClOHPA or 3,5‐DCP and different Cba lower ligand precursors (Table 1). No substantial change in the conversion rate of both substrates was detected, which strongly suggested that all the different Cbas were utilized by RdhA6 and RdhA3 for enzyme function.

**Table 1:** Reductive dehalogenation rates of different organohalides measured with crude extracts of *D. hafniense* DCB‐2 containing the RdhA6 or the RdhA3 enzyme

Amendment	RdhA6 activity (nkat mg^−1^)	RdhA3 activity (nkat mg^−1^)
No add.	0.89 ± 0.18	0.31 ± 0.09
DMB	1.67 ± 0.72	0.46 ± 0.26
5‐MeBza	1.26 ± 0.28	n.d.
Bza	1.10 ± 0.10	n.d.
5‐OHBza	1.27 ± 0.18	0.50 ± 0.27
5‐OMeBza	1.55 ± 0.23	0.35 ± 0.15
Purine	1.00 ± 0.09	0.21 ± 0.04
5‐azaBza	0.79 ± 0.10	n.d.
4‐azaBza	1.08 ± 0.17	n.d.
DMI	2.03 ± 0.27	0.40 ± 0.18
Imidazole	1.22 ± 0.22	n.d.

The average values of at least two independent cultivation experiments are given with the standard deviation (SD). DMB = 5,6‐dimethylbenzimidazole, 5‐MeBza = 5‐methylbenzimidazole, Bza = benzimidazole, 5‐OHBza = 5‐hydroxybenzimidazole, 5‐OMeBza = 5‐methoxybenzimidazole, 5‐azaBza = 5‐azabenzimidazole, 4‐azaBza = 4‐azabenzimidazole, DMI = 4,5‐dimethylimidazole, n.d. = not determined.

**Figure 2:**
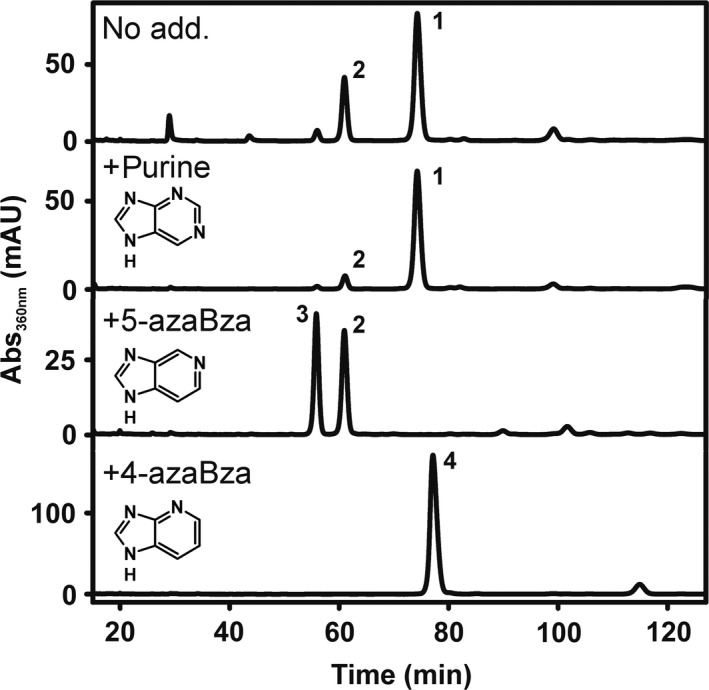
HPLC analysis of Cba extracts from *D. hafniense* DCB‐2 cells cultivated with pyruvate and ClOHPA in the presence of purine, 5‐azabenzimidazole (5‐azaBza), or 4‐azabenzimidazole (4‐azaBza) (25 μM respectively).

Based on the identification of unsubstituted purine or 5‐azaBza as lower base of Cbas produced in *D. hafniense* strain DCB‐2 and on the observation that the organism is susceptible to guided Cba biosynthesis with exogenous lower base precursors, we tested whether exogenous purine or azaBzas are also used as lower ligand precursors and steer Cba production. For this purpose, *D. hafniense* DCB‐2 was cultivated with pyruvate and ClOHPA in the presence of either purine, 5‐azaBza or 4‐azaBza. The Cbas were purified and analysed via HPLC coupled to photometric detection (Fig. 2) and ESI‐HR‐MS/MS (Tables S9–S12). As depicted in Figure 2, in cultures grown with purine amendment, the formation of the 5‐azaBza‐Cba was suppressed while the purinyl‐Cba was dominant; when 5‐azaBza was added, the purinyl‐Cba was not detected. These results indicate that exogenous sources of either purine or 5‐azaBza are efficiently exploited by *D. hafniense* strain DCB‐2 to cover the need for Cba lower ligand bases. Two Cbas (**2** and **3** in Fig. 2) with identical molecular formulas (Tables S10 and S11) were identified in cells cultivated in the presence of 5‐azaBza, which indicates the formation of two Cba isomers with the azaBza‐moiety most probably incorporated in two different orientations. The NMR analysis identified 5‐azaBza‐Cba in the case of compound **2;** thus, it is feasible that compound **3** represents 6‐azaBza‐Cba. Exogenous 4‐azaBza was also incorporated; however, in this case only a single isomer (**4** in Fig. 2) appeared to be formed and it was not investigated further, whether 4‐azaBza‐Cba or 7‐azaBza‐Cba was produced. The amendment of purine, 5‐azaBza or 4‐azaBza had no substantial effect on RdhA6 enzyme activity in crude extracts (Table 1).

**Figure 3:**
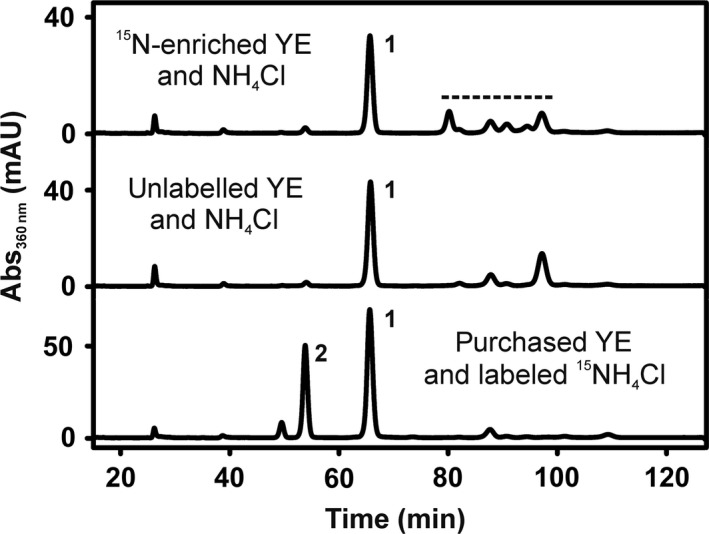
HPLC analysis of Cba extracts from *D. hafniense* strain DCB‐2 cultivated with pyruvate and ClOHPA in the presence of YE that was obtained from either ^15^N‐enriched or unlabelled *Saccharomyces cerevisiae* cells or purchased. For details about the production of the ^15^N‐enriched YE or the unlabelled YE, see Materials and Methods section. The ^15^N‐enriched YE or the unlabelled YE was added to *D. hafniense* strain DCB‐2 cultures that contained unlabelled NH_4_Cl as nitrogen source. When the purchased YE was applied, the unlabelled NH_4_Cl was replaced by ^15^N‐labelled NH_4_Cl (lower trace). The dashed line marks additional Cba signals. **1 **=** **purinyl‐Cba, **2 **=** **5‐azaBza‐Cba.

The finding of azaBza‐Cbas in cultures of *D. hafniense* strain DCB‐2 that were not amended with exogenous Cba lower base precursors raised the question for their origin. Genes for the known pathways of benzimidazole biosynthesis (Taga *et al*., 2007; Hazra *et al*., 2015) are not present in the genome of the organism (Kim *et al*., 2012). Strain DCB‐2 was routinely cultivated in medium containing 0.2% YE (purchased from Sigma‐Aldrich, Munich, Germany). Crofts *et al*. (2014b) reported the presence of benzimidazoles (pmol g^−1^) in YE. To unravel whether 5‐azaBza or purine or a precursor thereof was taken up from YE by the cells, a ^15^N‐isotopically labelled YE (^15^N‐enriched YE) was prepared and fed to *D. hafniense* strain DCB‐2 cultures. *Saccharomyces cerevisiae* was cultivated in the presence of ^15^NH_4_Cl, and from these cells, the ^15^N‐enriched YE was obtained. For comparison, unlabelled YE was produced from *S. cerevisiae* cells cultivated with non‐isotopically labelled NH_4_Cl. In a separate cultivation, the commercially available YE (purchased YE) was added to *D. hafniense* strain DCB‐2 in combination with ^15^N‐enriched NH_4_Cl. The HPLC elution profile of Cbas purified from cells cultivated in the presence of the produced YE differed from the profile obtained for the Cbas extracted from cells cultivated in the presence of the purchased YE (Fig. 3). The amount of 5‐azaBza‐Cba (**2**) was much lower compared to the reference sample, which strongly pointed towards the purchased YE as source of 5‐azaBza. Purinyl‐Cba (**1**) was identified as dominating Cba component by mass analysis in samples from cells cultivated with the YEs produced in the laboratory or with purchased YE. Comparative HR‐MS/MS analysis revealed ^15^N enrichment for both conditions with [^15^N_7_]‐purinyl‐Cba dominating for the application of ^15^N‐enriched YE and [^15^N_9_]‐purinyl‐Cba dominating for ^15^NH_4_Cl (Fig. S8A–C). While the exact location of the ^15^N labels within the corrin ring and the linker unit could not be unambiguously determined, ESI‐HR‐MS/MS analysis revealed that the purine unit is uniformly ^15^N‐labelled upon application of ^15^N‐enriched YE, but did not show any ^15^N enrichment upon growth in ^15^NH_4_Cl‐containing medium. These results demonstrate that the purinyl unit is obtained from the YE under the growth conditions applied here and that the 5‐azaBza‐unit or a precursor thereof might also be of exogenous origin. When the ^15^N‐enriched YE was applied, additional Cbas were detected (Fig. 3, signals are marked by a dashed line). ESI‐HR‐MS/MS analyses suggested the presence of adenine (C_8_H_9_N_2_, Fig. S9 and Table S14), guanine (C_5_H_5_N_5_O, Fig. S10 and Table S15), methylguanine (C_6_H_7_N_5_O, Fig. S11 and Table S16), methylhypoxanthine (C_6_H_6_N_4_O, Fig. S12 and Table S17) and dimethylimidazole (C_5_H_8_N_2_, Fig. S13 and Table S18) as lower ligands in these previously unknown Cbas.

**Figure 4:**
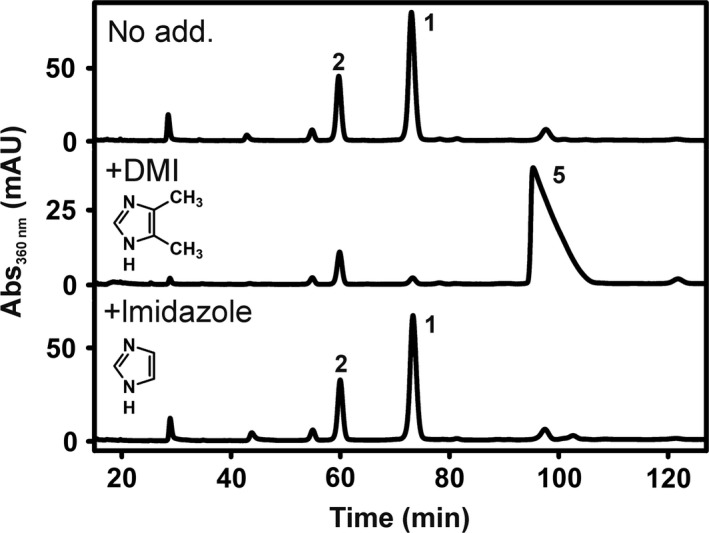
HPLC analysis of Cba extracts from *D. hafniense* strain DCB‐2 cultivated with pyruvate and ClOHPA in the presence of 4,5‐dimethylimidazole (DMI) or imidazole (25 μM respectively). **1 **=** **purinyl‐Cba, **2 **=** **5‐azaBza‐Cba.

In order to verify the utilization of exogenous dimethylimidazole as putative Cba lower base precursor by *D. hafniense* strain DCB‐2, the organism was cultivated in the presence of 4,5‐dimethylimidazole (DMI) (25 μM). The extraction of the Cbas from such cells and the subsequent analysis via HPLC‐ESI‐HR‐MS/MS uncovered the production of DMI‐Cba (**5**) (Fig. 4, Table S19). The utilization of unsubstituted imidazole for guided Cba biosynthesis in *Propionibacterium shermanii* was reported before by Kräutler *et al*. (1994). However, in the study presented here, unsubstituted imidazole was not incorporated into Cbas produced by *D. hafniense* strain DCB‐2. No negative effect on the RdhA6 activity in crude extracts of the organism was observed neither for DMI nor for imidazole.

### Impact of guided Cba biosynthesis on the heterologous production of *Desulfitobacterium* RDases

**Figure 5:**
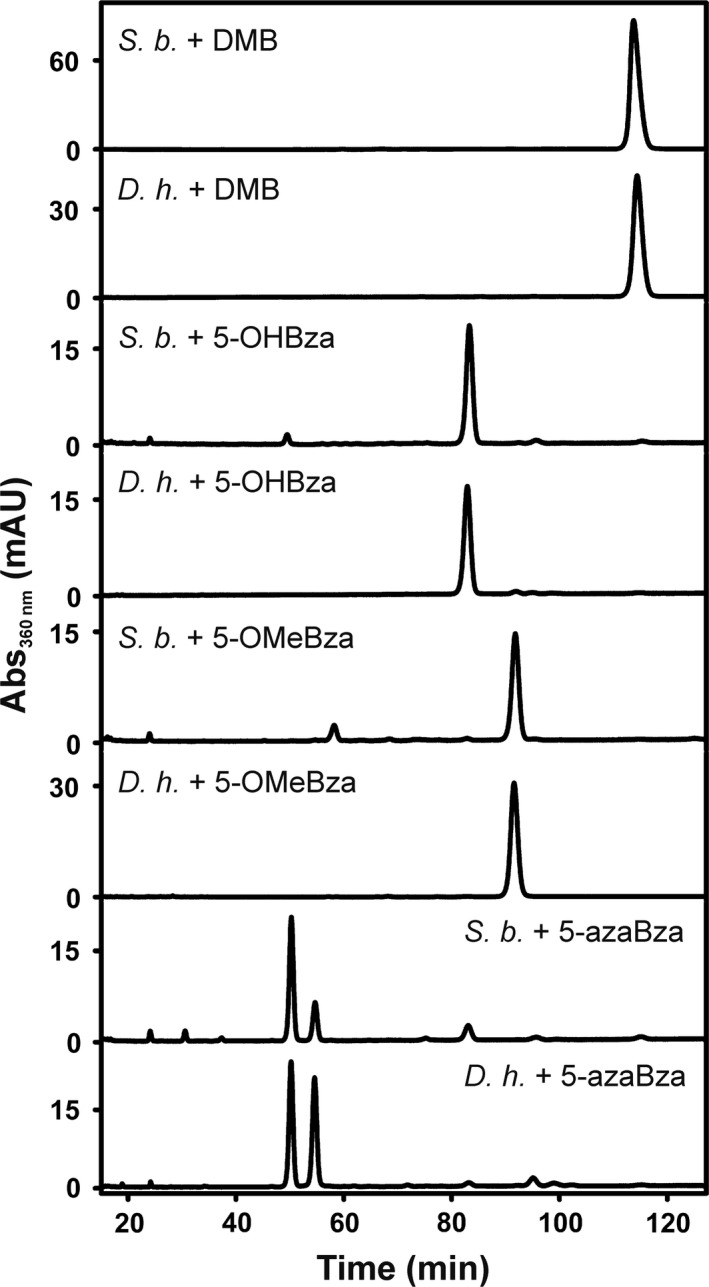
HPLC elution profiles of Cbas extracted from *S. blattae* (*S. b*.) in comparison with Cba samples derived from *D. hafniense* strain DCB‐2 (*D. h*.) cultivated in the presence of the same Bza.

In order to extend the analysis of Cba utilization by RDases originating from different *Desulfitobacterium* species including other *D. hafniense* strains or the heterologous host for *Desulfitobacterium* RDase production, the Cba‐producing *Shimwellia blattae* was analysed for guided Cba biosynthesis. *S. blattae* was cultivated under anoxic conditions with glycerol as growth substrate. Under these conditions, the organism performs glycerol fermentation that depends on the Cba‐containing glycerol dehydratase (Andres *et al*., 2004). It was shown in a previous study that *S. blattae* produces adeninyl‐Cba (pseudo‐B_12_) in the absence of exogenous DMB and DMB‐Cba when DMB was added to *S. blattae* cultures (Mac Nelly *et al*., 2014). Here, these initial tests were extended by analysing other Bzas for their utilization as Cba lower bases by *S. blattae* (Fig. 5). The comparison of HPLC elution profiles of Cba extracts from *S. blattae* and *D. hafniense* DCB‐2 indicated the incorporation of 5‐OHBza, 5‐OMeBza and 5‐azaBza, respectively, into Cbas formed by *S. blattae*.

**Figure 6:**
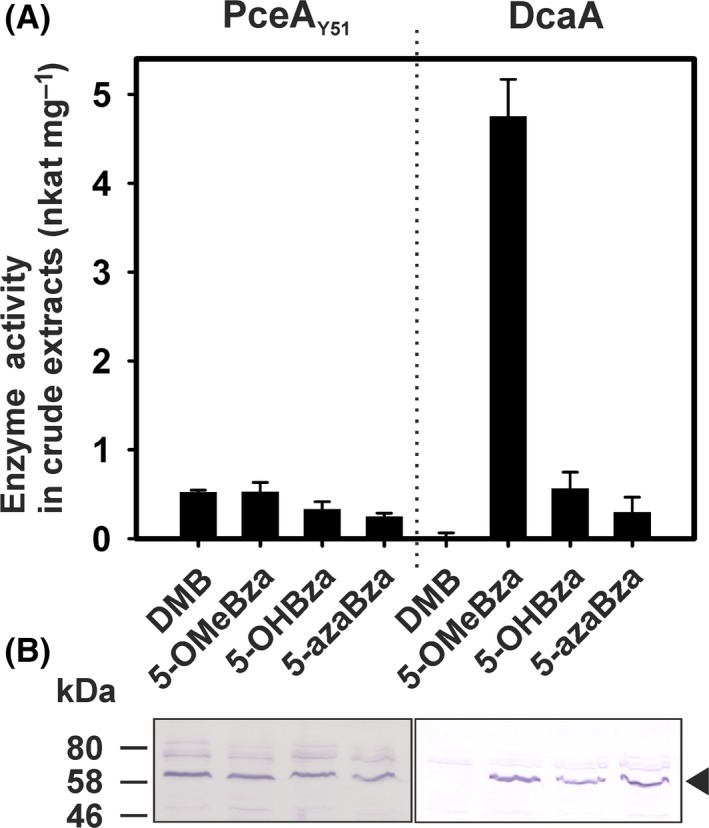
Enzyme activity and protein level of PceA_Y51_ and DcaA heterologously produced in *S. blattae* cultivated in the presence of various amendments. A. The RDase activity was measured in at least two independent cultures. The standard deviation is given. PceA_Y51_ was tested for the conversion of PCE, while DcaA was tested with 1,2‐dichloroethane (DCA) as substrate. B. Immunological detection of both RDases in crude extracts separated on an SDS‐PAGE (25 μg of protein was applied to each lane) with an antibody directed against the Strep‐tag.

To evaluate the impact of Cba guided biosynthesis on the formation of catalytically active RDase enzymes in *S. blattae*, the tetrachloroethene RDase of *D. hafniense* strain Y51 (PceA_Y51_) and the 1,2‐dichloroethane RDase of *Desulfitobacterium dichloroeliminans* (DcaA) were produced in the organism and tested for substrate conversion under the different conditions (Fig. 6). These enzymes were chosen because PceA_Y51_ and DcaA display an elevated sequence identity to RdhA3 of *D. hafniense* strain DCB‐2 (68% and 67% respectively) and might share the same cobamide cofactor preference. While strain *S. blattae* (Strep‐*pceApdcaT*) was used for the production of PceA_Y51_, the production of DcaA was conducted with strain *S. blattae* (Strep‐*dcaApdcaT*) (Kunze *et al*., 2017). In the case of PceA_Y51_ a PCE‐dechlorinating enzyme activity of about 0.5 nkat mg^−1^ was measured when DMB was added to the cultures, which was in accordance with previous results. The activity of PceA_Y51_ was not changed in the presence of the alternative lower base precursor 5‐OMeBza (Fig. 6A) and was slightly reduced when 5‐OHBza or 5‐azaBza was present. The amount of PceA_Y51_ did not change in the differentially treated cells as monitored via immunological detection (Fig. 6B). In the case of DcaA, a different result was obtained. When DMB was added, DcaA was not detectable in the cells. Extracts obtained from cells cultivated in the presence of 5‐OHBza and 5‐azaBza mediated the dihaloelimination of 1,2‐dichloroethane to ethene by DcaA, and the protein was detected in the immunoblot. A substantial increase (about 10‐fold) in the conversion rate was observed when 5‐OMeBza instead of 5‐OHBza or 5‐azaBza was applied. Since this effect was not accompanied by an increase in the DcaA protein level under these conditions or could have been explained by a substantial change in the Cba content of the *S. blattae* cells cultivated in the presence of 5‐OMeBza, 5‐OHBza or 5‐azaBza (SD = 11%), these results implied either an increased specific enzyme activity of DcaA or an elevated efficiency of the cofactor incorporation.

### Cobamide cofactor content of heterologously produced DcaA

In order to determine the Cba cofactor content, DcaA was purified from the soluble extract of cells cultivated in the presence of 5‐OMeBza or 5‐azaBza and the Cba content was analysed. In the case of cells cultivated in the presence of 5‐OMeBza, only about 5% of the purified protein complex composed of DcaA and its dedicated chaperone DcaT (a 1:1 stoichiometry was assumed) contained a Cba cofactor (Fig. S14). The amount of Cba extracted from the enzyme purified from 5‐azaBza‐treated cells was substantially lower and close to the detection limit, a result that further supported the conclusion of a preferential use of the 5‐OMeBza‐containing Cba as cofactor by DcaA. Based on this result, efforts were made to reconstitute the purified DcaA enzyme with 5‐OMeBza‐Cba and other Cbas, but until today no successful reconstitution was achieved. The preference of DcaA for 5‐OMeBza‐Cba was not reported for the PCE‐RDase of *S. multivorans* (Keller *et al*., 2018), although both enzymes share the sensitivity to DMB‐containing Cba cofactors. The different behaviour towards 5‐OMeBza‐Cba might be explained by the low sequence identity between both enzymes. However, even between enzymes that share a high sequence similarity (87.5% identity and 93% similarity between PceA_Y51_ and DcaA when analysed without the signal peptide for membrane export), differences in the cofactor preference have been observed here. So far, the molecular basis for the Cba cofactor preferences of selected RDases remains elusive. A systematic mutagenesis approach is needed for the identification of amino acid residues that might function as decisive structural elements in Cba incorporation.

## Discussion

The presence of yeast extract (YE) can have a promoting effect on anaerobic reductive dehalogenation in bioreactors (Hendriksen and Ahring, 1992). The addition of YE to culture media of reductively dehalogenating *Desulfitobacterium* spp. was frequently reported before (Utkin *et al*., 1994; Gerritse *et al*., 1997; Miller *et al*., 1997; Krasotkina *et al*. 2001; Suyama *et al*., 2001; De Wildeman *et al*., 2003). However, YE might be a dispensable amendment for the cultivation of *Desulfitobacterium* spp. (Sanford *et al*., 1996; Yan *et al*., 2018). In the actual study, YE was routinely added in order to investigate the impact of undefined medium components on cobamide (Cba) biosynthesis in the organism. As shown here, YE served as a reservoir of Cba lower ligand bases for *D. hafniense* strain DCB‐2. In the presence of YE and in the absence of defined exogenous lower base precursors, the organism produced different Cbas including azaBza‐Cbas, Cbas containing imidazole derivatives as lower base and the purinyl‐Cba. The purinyl moiety of the latter was recruited from YE as has been shown by the ^15^N‐labelling experiment. This finding points out the role of undefined medium components as a potential source of Cba lower ligand precursors. Such precursors (Bzas, purines or imidazoles) might be taken up by the cells, alter the composition of the Cba cofactor pool and can negatively or positively interfere with the function of a Cba‐containing enzyme. In order to generate a homogeneous pool of Cba cofactors inside a Cba‐producing organism, a distinct Cba lower ligand precursor might be added to the growth medium as it has been performed in this study.

Purines and other Cba lower ligand precursors are channelled into Cba biosynthesis by the nicotinate‐benzimidazole phosphoribosyltransferase (CobT) (Trzebiatowski *et al*., 1994; Crofts *et al*., 2013; Hazra *et al*., 2013). The *cobT* gene product (GenBank: ACL21303.1) in *D. hafniense* strain DCB‐2 shares 100% sequence identity to its homologue (GenBank: BAE83903.1) in *D. hafniense* strain Y51. Based on this sequence match, a similar preference in the utilization of lower base precursors for Cba biosynthesis in both strains was assumed, and indeed, the production of purinyl‐Cba in *D. hafniense* strain DCB‐2 was observed. Imidazoles might also be channelled into Cba biosynthesis by CobT homologues (Trzebiatowski and Escalante‐Semerena, 1997) and should be taken into consideration, when the limits of enzyme‐Cba cofactor specificity are investigated. In the study presented here, the incorporation of 4,5‐dimethylimidazole (DMI) as lower base into Cbas by *D. hafniense* strain DCB‐2 was shown and the biosynthesis of DMI‐Cba did not interfere with RDase function.

Guided Cba biosynthesis is an efficient tool to change the structure of Cba cofactors in Cba‐producing bacteria and to investigate cofactor selectivity of Cba‐containing enzymes such as reductive dehalogenases (RDases) in the native organism or the host for heterologous production. Here, the Cba‐dependent enzymatic reductive dehalogenation of ClOHPA by RdhA6 and 3,5‐DCP by RdhA3 of *D. hafniense* strain DCB‐2 was shown to be unaffected by guided Cba biosynthesis that led to the incorporation of purine, 5‐azaBza, 4‐azaBza, DMB, MeBza, Bza, OHBza or OMeBza as lower base into the cofactor. Although the efficient incorporation of the various Cbas into both RDases was not proven, the results strongly implied a flexibility of RdhA6 and RdhA3 in Cba utilization and led to the conclusion that both enzymes are versatile rather than specific in this respect.

In the current decade, evidence was obtained that variations in the structure of Cba cofactors can interfere with the enzymatic reductive dehalogenation in organohalide‐respiring bacteria (OHRB), which pointed towards specific limitations in Cba utilization by RDases (Yan *et al*., 2012, 2013, 2016; Yi *et al*., 2012; Keller *et al*., 2014, 2018). For example, the RDase‐mediated dehalogenation of tetrachloroethene (PCE) in the epsilonproteobacterium *Sulfurospirillum multivorans* was shown to function with norcobamide (NCbas) cofactors containing adenine, OHBza or OMeBza as lower ligand base, but was affected to different extent when DMB or MeBza was the lower base (Keller *et al*., 2014, 2018). The molecular basis for this specificity has not been clearly assigned yet, but a negative interference of both NCbas with the process of cofactor incorporation into the cytoplasmic precursor of the PCE‐RDase (PceA) was proposed (Keller *et al*., 2018). From the structural information available for RDases, it was inferred that the nucleotide loop including the lower base of the Cba plays a role in the cofactor binding rather than in the Cba‐mediated enzymatic reaction (Bommer *et al*., 2014; Payne *et al*., 2015). The preferences in Cba utilization observed for selected RDases such as PceA of *S. multivorans* (Keller *et al*., 2018) or DcaA of *D. dichloroeliminans* (this study) could reflect an adaptation to the predominance of a specific Cba in the evolutionary history of the enzyme. In contrast, the observed versatility in the Cba utilization of RdhA6 and RdhA3 of *D. hafniense* strain DCB‐2 might indicate the permanent availability of a broad range of Cba cofactors to both enzymes in the past.

The mechanism of Cba incorporation into RDases and the exact positioning of the cofactors inside the enzymes investigated in this study remain unknown. Hence, the observed specificity of DcaA of *D. dichloroeliminans* for OMeBza‐Cba cannot be explained at the moment. Structural models of PceA_Y51_ of *D. hafniense* strain Y51 and DcaA, which were generated on the basis of the three‐dimensional structure of PceA from *S. multivorans* (Bommer *et al*., 2014), showed that the Cba cofactor is bound in the base‐off mode inside the enzymes (Kunze *et al*., 2017). Given the fact that PceA_Y51_ and DcaA have a high similarity in amino acid sequence and that the Cba cofactor‐binding core is structurally conserved among RDases (Bommer *et al*., 2014; Payne *et al*., 2015), it is feasible that only minimal structural changes are sufficient to generate Cba preferences as displayed by the comparison of PceA_Y51_ and DcaA heterologously produced in *S. blattae*. The identification of these structural elements in *Desulfitobacterium* RDases should be the goal of further studies, since it is expected to allow for the improvement of the platform for heterologous production of these environmentally and biotechnological relevant biocatalysts.

## Experimental procedures

### Cultivation of bacterial strains

*Desulfitobacterium hafniense* strain DCB‐2 (DSM No. 10664^T^) was cultivated in a medium (Scholz‐Muramatsu *et al*., 1995; Reinhold *et al*., 2012) amended with 0.2% (w/v) yeast extract (order no. 92144, lot no. BCBH5306V, Sigma‐Aldrich, Munich, Germany). Vitamins were added as described in Scholz‐Muramatsu *et al*., (1995), but vitamin B_12_ was not included. Pyruvate (40 mM) was present as electron donor and carbon source. For the induction of RDase gene expression in *D. hafniense* DCB‐2, either 3‐chloro‐4‐hydroxy‐phenylacetate (ClOHPA) (10 mM) or 3,5‐dichlorophenol (3,5‐DCP) (100 μM) was added as electron acceptor. ClOHPA was transferred from an anoxic and sterile solution (0.5 M) in ultrapure water (UPW), while 3,5‐DCP was added from an anoxic sterile stock solution (0.1 M) prepared in ethanol. The inoculum was 10% of the culture volume. For the cultivation with 3,5‐DCP, a preculture fermentatively grown on pyruvate was used. Cells were harvested for further analyses after three passages on a particular medium composition. In the case of cultures cultivated with ClOHPA, the cells were harvested (10 min, 12 000 × *g*, 4°C) after 24 h, in the case of 3,5‐DCP, the cells were sedimented by centrifugation after 48 h. While ClOHPA was added prior to the inoculation, 3,5‐DCP was added after a 24 h cultivation period in the absence of the halogenated substrate. Purine, 4,5‐dimethylimidazole (DMI), imidazole or the various benzimidazoles were added from anoxic sterile stock solutions (4 mM) in UPW. If not stated otherwise, all chemical compounds were purchased from Sigma‐Aldrich (Munich, Germany). 5‐hydroxybenzimidazole was purchased from Combi‐Blocks (San Diego, CA, USA), and DMI from Santa Cruz Biotechnology (Dallas, TX, USA).

The transcript level determination of *rdhA6* of *D. hafniense* strain DCB‐2 was conducted as reported before (Mac Nelly *et al*., 2014). *Shimwellia blattae* (ATCC No. 33430) and the RDase‐producing *S. blattae* mutant strains were cultivated as reported previously (Kunze *et al*., 2017).

### Production of a [^15^N]‐enriched yeast extract (YE)

Fed‐batch cultivations of *Saccharomyces cerevisiae* were performed in a fermenter (volume 1 l, BioFlo C30, New Brunswick) with 750 ml ultrapure water (UPW) containing 2.5 g K_2_SO_4_, 1.1 g MgSO_4_ × 7 H_2_O, 2.07 g NaH_2_PO_4_, 3.4 g NH_4_Cl and 0.5 g YE. The nutrient solution (250 ml) contained 37.5 g glucose and 5 g NH_4_Cl. For the generation of the [^15^N]‐enriched YE, NH_4_Cl was replaced by ^15^NH_4_Cl (Sigma‐Aldrich, Munich, Germany). The initial OD (578 nm) was 0.5‐0.6. After 1 day of cultivation, about 25 g of wet cells (OD_578nm_ = 12–13) was harvested. From *S. cerevisiae* cell pellets obtained from cultivations with either unlabelled NH_4_Cl or labelled ^15^NH_4_Cl, yeast extracts were produced in accordance to a method published by Opitz *et al*. (2015). The wet cells were resuspended in 25 ml UPW. The pH was adjusted to 7.5 by the dropwise addition of 10 N NaOH. Lyophilized powder of lyticase (Sigma‐Aldrich, Munich, Germany) from *Arthrobacter luteus* was added to the cell suspension (in total 12.5 kU). The mixture was stirred at 37°C. The pH was adjusted to 7.5 after 90, 180 and 270 min of incubation time. After overnight incubation, the cell suspension was passed through a French Press (SLM AMINCO Spectronic Instruments, Rochester, USA) at 1000 Psi, to ensure complete destruction of the cells. The crude cell‐free extract was incubated at 65°C for 20 min. The pH was adjusted to 6.0, and 0.5% (w/v) papain was added. The mixture was stirred at 50°C for 5 days. Subsequently, the cell‐free extract was manually homogenized with a glass tissue homogenizer. The suspension was centrifuged for 1 h at 20 000 rpm in a JA‐20 rotor of an Avanti JXN‐26 centrifuge (Beckman‐Coulter, Krefeld, Germany) and for 45 min at 36 000 rpm in a Ti 70 rotor of a L8‐70M ultracentrifuge (Beckman‐Coulter, Krefeld, Germany). The supernatant was freeze‐dried in a LYOVAC GT 2 lyophilizer (FINN‐AQUA Santasalo‐Sohlberg Hürth, Germany). About 5 g of YE was obtained in both cases, resuspended in 11.5 ml UPW (final volume), transferred into a serum bottle, made anoxically by alternating evacuation and N_2_‐flushing of the gas phase and autoclaved. Due to the use of an unlabelled inoculum and the presence of unlabelled YE in the medium for the *S. cerevisiae* cultivation, about 7% of the ^15^N‐enriched YE produced in this experiment did not contain ^15^N.

### Cobamide purification and analysis

The purification of the cobamides (Cbas) was conducted as described earlier (Keller *et al*., 2014). For the analysis of the Cbas by high‐performance liquid chromatography (HPLC), a Kinetex 5 μm EVO C18 100 Å LC Column 250 × 4.6 mm (Phenomenex, Aschaffenburg, Germany) was applied. The separation was performed isocratically with 14% methanol/0.2% acetic acid at 30°C and with a flow rate of 0.5 ml min^−1^. The UV/Vis‐absorbance spectra of the purified Cbas were recorded with a Cary 100 UV‐visible spectrophotometer (Agilent Technologies, Waldbronn, Germany).

### Liquid Chromatography–Electrospray Ionization–High‐Resolution Tandem Mass Spectrometry (HPLC‐ESI‐HR‐MS/MS) of cobamides

HPLC‐ESI‐HR‐MS/MS analysis of Cba‐containing fractions was performed using a Dionex UltiMate 3000 HPLC instrument coupled to a Bruker Maxis high‐resolution qTOF mass spectrometer equipped with an electrospray ionization (ESI) unit operated in positive mode. Chromatographic separations were achieved using an Agilent ZORBAX Eclipse XDB‐C18 column (250 × 3 mm, 5 μm particle diameter) with a flow rate of 400 μl min^−1^ and gradient elution starting at 3% acetonitrile in 0.5% aqueous acetic acid for 5 min followed by a linear increase to 100% acetonitrile with 0.5% acetic acid within 35 min. The ESI‐(+)‐MS spectra of Cbas show signals for the [M+H]^+^ and the [M + 2H]^2+^ adducts. HR‐MS/MS analysis of the [M+H]^+^ precursor ion upon fragmentation with CID 70 affords common fragment ions at *m/z* 1209.4903 [C_54_H_79_CoN_12_O_14_P]^+^ due to loss of the heteroaromatic ligand, *m/z* 997.4817 [C_49_H_70_CoN_12_O_7_]^+^ due to loss of the phosphoriboside unit, as well as *m/z* 1124.4501 [C_51_H_74_CoN_10_O_13_P]^+^ and *m/z* 912.4415 [C_46_H_65_CoN_10_O_6_]^+^ due to subsequent loss of a C_3_H_5_N_2_O unit (−85.0402 amu), previous assignment of this fragmentation as loss of CoCN (−84.9363 amu) could not be confirmed by the HRMS measurements. Furthermore, HR‐MS/MS analysis of the doubly charged [M + 2H]^2+^ precursor ion upon fragmentation with CID 30 affords compound‐specific fragment ions for the phosphoriboside unit and the free base ligand, along with the common fragments obtained from the [M+H]^+^ precursor.

Consequently, known and yet unidentified Cbas were detected by HR‐MS/MS analysis using an automated precursor ion selection routine optimized for [M + 2H]^2+^ ions in the range of *m/z* 600–700 along with a CID energy of 30. Putative Cbas were detected by screening for characteristic MS/MS fragment ions at *m/z* 1209.5 (±0.1) corresponding to [M – Ligand]^+^. Molecular formulas of Cbas were determined based on the [M+H]^+^ and [M + 2H]^2+^ signals, and the identification of the compound‐specific lower base was subsequently confirmed by inspection of the corresponding [base + H]^+^ and [phosphoriboside + H]^+^ fragments.

### Isolation of cobamides for NMR analysis

For ^1^H‐NMR analyses, the Cbas were further purified via solid phase extraction on a CHROMABOND^®^ HR‐X column (3 mL, 200 mg; Macherey‐Nagel, Düren, Germany) according to the manufacturer's instructions. Purinyl cobamide (**1**) and 5‐azabenzimidazolyl cobamide (**2**) were isolated from the cobamide extracts by semipreparative HPLC using an Agilent HP‐1100 HPLC instrument equipped with a Grom‐Sil 120 ODS‐4 HE column (250 × 8 mm, 5 μm) coupled to a Gilson 206 Abimed fraction collector. A flow rate of 2 ml min^−1^ with gradient elution was used starting at 3% acetonitrile in 0.5% aqueous acetic acid for 3 min, followed by a linear increase to 100% acetonitrile with 0.5% acetic acid within 30 min. Fractions were analysed by HPLC‐MS, concentrated under reduced pressure and dried under vacuum to afford approximately 150 μg purinyl cobamide (**1**) and 150 μg 5‐azabenzimidazolyl cobamide (**2**).

### NMR analysis

^1^H‐NMR, ^1^H,^1^H‐PRESAT‐*dqf*‐COSY, ^1^H,^13^C‐HSQC, ^1^H,^13^C‐HMBC and ^1^H,^1^H‐PRESAT‐ROESY spectra were recorded using a Bruker Avance III HD 700 spectrometer equipped with a 1.7 mm TCI microcryoprobe and a cryoplatform (Bruker Biospin GmbH, Rheinstetten, Germany). NMR tubes of 1.7 mm outer diameter were used. All NMR spectra were recorded at 298 K using D_2_O as a solvent. Spectrometer control was accomplished using Bruker TopSpin 3.2 software (Bruker Biospin). Standard pulse programs as implemented in TopSpin were used. For data processing, TopSpin 3.2 and ACD/Labs release 2012 (ACDLabs, Frankfurt, Germany) were used respectively. Prior to data acquisition, the spectrometer was carefully tuned to the transmitter frequencies for ^1^H, ^13^C and ^15^N. Chemical shifts were left uncorrected.

### RDase activity measurements, purification of DcaA and cofactor content determination

The RDase enzyme activity in crude extracts of *D. hafniense* strain DCB‐2 cells was measured photometrically with methyl viologen (reduced with titanium(III) citrate) as electron donor (Neumann *et al*., 1996). A 100 ml of culture was harvested under oxic conditions (10 min, 12 000 × *g*, 4°C). Transferred to an anoxic chamber, the cell pellet was resuspended in anoxic buffer (50 mM Tris‐HCl, pH 7.5; per gram wet cells 3 ml buffer). The cell suspension was mixed with an equal volume of glass beads (0.25–0.5 mm diameter, Carl Roth GmbH, Karlsruhe, Germany). The cells were disrupted using a beadmill (10 min at 30 Hz; Mixer Mill MM400, Retsch GmbH, Haan, Germany). Crude extract and glass beads were separated by mild centrifugation (2 min, 2000 × *g*). The supernatant (crude extract) was applied for the measurement of the enzyme activity. The concentration of ClOHPA or 3,5‐DCP was 1 mM in the enzymatic assay. Both compounds were added from stock solutions (100 mM) in ethanol. Measurements of RDase (PceA_Y51_ or DcaA) enzyme activity in crude extracts of *S. blattae* mutant strains, purification of recombinant DcaA, and immunological detection of StrepDcaA were conducted in accordance to protocols described by Kunze *et al*. (2017). Protein concentrations were determined by the Bradford assay (1976).

## Conflict of interest

None declared.

## Supporting information


**Fig. S1.** UV/Vis‐absorbance spectra of the purified Cbas from *Desulfitobacterium hafniense* strain DCB‐2.
**Fig. S2.** MS/MS fragmentation of cobamides using [M+H]^+^ and [M+2H]^2+^ precursor ions with a CID energy of 70 and 30, respectively.
**Fig. S3:** Section of the HMBC spectrum of the purinyl cobamide (**1**) isolated from *D. hafniense* strain DCB‐2 showing H,C‐correlations in the purine unit.
**Fig. S4:** Section of the HMBC spectrum of the 5‐azabenzimidazolyl cobamide (**2**) isolated from *D. hafniense* strain DCB‐2 showing H,C‐correlations in the 5‐azabenzimidazole unit.
**Fig. S5:** Orientation of the heteroaromatic ligands as deduced from analysis of NOE‐correlations observed in the 700 MHz ROESY spectrum.
**Fig. S6.** Low field range of ^1^H‐NMR spectra of 5‐MeBza‐Cba, 6‐OHBza‐Cba and 5‐OHBza‐Cba, and 5‐OMeBza‐Cba.
**Fig. S7.** Relative transcript levels of the *rdhA1*,* rdhA3*,* rdhA4*,* rdhA5*, and *rdhA6* genes in two cultures of *D. hafniense* strain DCB‐2.
**Fig. S8** A: HR‐MS/MS analysis of the purinyl cobamide (**1**) from *D. hafniense* strain DCB‐2 supplemented with [^15^N]‐enriched yeast extract or [^15^N]‐enriched NH_4_Cl. B: HR‐MS/MS analysis of the purinyl cobamide (**1**) from *D. hafniense* strain DCB‐2 supplemented with [^15^N]‐enriched yeast extract or [^15^N]‐enriched NH_4_Cl. C: HR‐MS/MS analysis of the purinyl cobamide (**1**) from *D. hafniense* strain DCB‐2 supplemented with [^15^N]‐enriched yeast extract or [^15^N]‐enriched NH_4_Cl.
**Fig. S9:** HPLC‐ESI‐(+)‐HR‐MS chromatogram of [M+2H]^2+^ signals corresponding to putative adeninyl cobamides from *D. hafniense* strain DCB‐2 supplemented with the ^15^N‐enriched YE.
**Fig. S10:** HPLC‐ESI‐(+)‐HR‐MS chromatogram of [M+2H]^2+^ signals corresponding to putative guaninyl cobamides from *D. hafniense* strain DCB‐2 supplemented with the ^15^N‐enriched YE.
**Fig. S11:** HPLC‐ESI‐(+)‐HR‐MS chromatogram of [M+2H]^2+^ signals corresponding to putative methylguaninyl cobamides from *D. hafniense* strain DCB‐2 supplemented with the ^15^N‐enriched YE.
**Fig. S12:** HPLC‐ESI‐(+)‐HR‐MS chromatogram of [M+2H]^2+^ signals corresponding to putative methylhypoxanthinyl cobamides from *D. hafniense* strain DCB‐2 supplemented with the ^15^N‐enriched YE.
**Fig. S13:** HPLC‐ESI‐(+)‐HR‐MS chromatogram of [M+2H]^2+^ signals and HPLC‐ESI‐(+)‐HR‐MS/MS data of dimethylimidazolyl cobamide from *D. hafniense* strain DCB‐2 supplemented with the ^15^N‐enriched YE.
**Fig. S14:** Purification of recombinant Strep‐DcaA. The soluble fractions (10 µg protein) and the eluates (1 µg protein) were separated on a 12.5% SDS/PAGE (Coomassie‐stained).
**Table S1.** HPLC‐ESI‐(+)‐HR‐MS/MS data of a vitamin B_12_ standard.
**Table S2.** HPLC‐ESI‐(+)‐HR‐MS/MS data of the purinyl cobamide (signal **1** in Fig. 1) from *D. hafniense* strain DCB‐2 supplemented with YE, but without other additives.
**Table S3.** HPLC‐ESI‐(+)‐HR‐MS/MS data of the 5‐azabenzimidazolyl cobamide (signal **2** in Fig. 1) from *D. hafniense* strain DCB‐2 supplemented with YE, but without other additives.
**Table S4.** HPLC‐ESI‐(+)‐HR‐MS/MS data of the 5,6‐dimethylbenzimidazolyl cobamide from *D. hafniense* strain DCB‐2 supplemented with YE and 5,6‐dimethylbenzimidazole (DMB).
**Table S5.** HPLC‐ESI‐(+)‐HR‐MS/MS data of the benzimidazolyl cobamide from *D. hafniense* strain DCB‐2 supplemented with YE and benzimidazole (Bza).
**Table S6.** HPLC‐ESI‐(+)‐HR‐MS/MS data of the 5‐methylbenzimidazolyl cobamide from *D. hafniense* strain DCB‐2 supplemented with YE and 5‐methylbenzimidazole (5‐MeBza).
**Table S7.** HPLC‐ESI‐(+)‐HR‐MS/MS data of the 5‐methoxybenzimidazolyl cobamide from *D. hafniense* strain DCB‐2 supplemented with YE and 5‐methoxybenzimidazole (5‐OMeBza).
**Table S8.** HPLC‐ESI‐(+)‐HR‐MS/MS data of the 5‐/6‐hydroxybenzimidazolyl cobamide(s) from *D. hafniense* strain DCB‐2 supplemented with YE and 5‐hydroxybenzimidazole (5‐OHBza).
**Table S9.** HPLC‐ESI‐(+)‐HR‐MS/MS data of the purinyl cobamide (**2**) from *D. hafniense* DCB‐2 supplemented with YE and purine.
**Table S10.** HPLC‐ESI‐(+)‐HR‐MS/MS data of the 5‐azabenzimidazolyl cobamide (**2**) from *D. hafniense* DCB‐2 supplemented with YE and 5‐azabenzimidazole (5‐azaBza).
**Table S11.** HPLC‐ESI‐(+)‐HR‐MS/MS data of the putative 6‐azabenzimidazolyl cobamide (**3**) from *D. hafniense* DCB‐2 supplemented with YE and 5‐azabenzimidazole (5‐azaBza).
**Table S12.** HPLC‐ESI‐(+)‐HR‐MS/MS data of the 4‐azabenzimidazolyl cobamide (**4**) from *D. hafniense* DCB‐2 supplemented with YE and 4‐azabenzimidazole (4‐azaBza).
**Table S13.** NMR data (700 MHz, D_2_O) for the purinyl cobamide (**1**) and the 5‐azabenzimidazolyl cobamide (**2**) isolated from *D. hafniense* strain DCB‐2.
**Table S14.** HPLC‐ESI‐(+)‐HR‐MS/MS data of putative adeninyl cobamides from *D. hafniense* strain DCB‐2 supplemented with the ^15^N‐enriched YE.
**Table S15.** HPLC‐ESI‐(+)‐HR‐MS/MS data of putative guaninyl cobamides from *D. hafniense* strain DCB‐2 supplemented with the ^15^N‐enriched YE.
**Table S16.** HPLC‐ESI‐(+)‐HR‐MS/MS data of putative methylguaninyl cobamides from *D. hafniense* strain DCB‐2 supplemented with the ^15^N‐enriched YE.
**Table S17.** HPLC‐ESI‐(+)‐HR‐MS/MS data of putative methylhypoxanthinyl cobamides from *D. hafniense* strain DCB‐2 supplemented with the ^15^N‐enriched YE.
**Table S18.** HPLC‐ESI‐(+)‐HR‐MS data of putative dimethylimidazolyl cobamide from *D. hafniense* strain DCB‐2 supplemented with the ^15^N‐enriched YE.
**Table S19.** HPLC‐ESI‐(+)‐HR‐MS/MS data of the 4,5‐dimethylimidazolyl cobamide (signal **5** in Fig. 4) from *D. hafniense* strain DCB‐2 supplemented with 4,5‐dimethylimidazole (DMI).
