# Supplementary material for: Guided cobamide biosynthesis for heterologous production of reductive dehalogenases
Source: Microb Biotechnol. 2018 Dec 13;12(2):346–59. doi: 10.1111/1751-7915.13339 (PMC6389850; doi:10.1111/1751-7915.13339)
Supplement: Supplementary file 1 — Fig. S1. UV/Vis‐absorbance spectra of the purified Cbas from Desulfitobacterium hafniense strain DCB‐2. Fig. S2. MS/MS fragmentation of cobamides using [M+H]+ and [M+2H]2+ precursor ions with a CID energy of 70 and 30, respectively. Fig. S3: Section of the HMBC spectrum of the purinyl cobamide (1) isolated from D. hafniense strain DCB‐2 showing H,C‐correlations in the purine unit. Fig. S4: Section of the HMBC spectrum of the 5‐azabenzimidazolyl cobamide (2) isolated from D. hafniense strain DCB‐2 showing H,C‐correlations in the 5‐azabenzimidazole unit. Fig. S5: Orientation of the heteroaromatic ligands as deduced from analysis of NOE‐correlations observed in the 700 MHz ROESY spectrum. Fig. S6. Low field range of 1H‐NMR spectra of 5‐MeBza‐Cba, 6‐OHBza‐Cba and 5‐OHBza‐Cba, and 5‐OMeBza‐Cba. Fig. S7. Relative transcript levels of the rdhA1, rdhA3, rdhA4, rdhA5, and rdhA6 genes in two cultures of D. hafniense strain DCB‐2. Fig. S8 A: HR‐MS/MS analysis of the purinyl cobamide (1) from D. hafniense strain DCB‐2 supplemented with [15N]‐enriched yeast extract or [15N]‐enriched NH4Cl. B: HR‐MS/MS analysis of the purinyl cobamide (1) from D. hafniense strain DCB‐2 supplemented with [15N]‐enriched yeast extract or [15N]‐enriched NH4Cl. C: HR‐MS/MS analysis of the purinyl cobamide (1) from D. hafniense strain DCB‐2 supplemented with [15N]‐enriched yeast extract or [15N]‐enriched NH4Cl. Fig. S9: HPLC‐ESI‐(+)‐HR‐MS chromatogram of [M+2H]2+ signals corresponding to putative adeninyl cobamides from D. hafniense strain DCB‐2 supplemented with the 15N‐enriched YE. Fig. S10: HPLC‐ESI‐(+)‐HR‐MS chromatogram of [M+2H]2+ signals corresponding to putative guaninyl cobamides from D. hafniense strain DCB‐2 supplemented with the 15N‐enriched YE. Fig. S11: HPLC‐ESI‐(+)‐HR‐MS chromatogram of [M+2H]2+ signals corresponding to putative methylguaninyl cobamides from D. hafniense strain DCB‐2 supplemented with the 15N‐enriched YE. Fig. S12: HPLC‐ESI‐(+)‐HR‐MS chromatogram of [M+2H]2+ signals corr [file MBT2-12-346-s001.pdf]

SUPPORTING INFORMATION

Guided cobamide biosynthesis for heterologous production of reductive dehalogenases

Torsten Schubert[1], Stephan H. von Reuß[2], Cindy Kunze[1], Christian Paetz[3], Stefan Kruse[1], Peggy Brand-Schön[1], Christoph Baum[1], Anita Mac Nelly[1], Jörg Nüske[1], and Gabriele Diekert[1]

[1]Department of Applied and Ecological Microbiology, Institute of Microbiology, Friedrich Schiller University, Philosophenweg 12, D-07743 Jena, Germany

[2]Department of Bioorganic Chemistry and [3]Research Group Biosynthesis / NMR, Max Planck Institute for Chemical Ecology, Hans-Knöll-Straße 8, D-07745 Jena, Germany

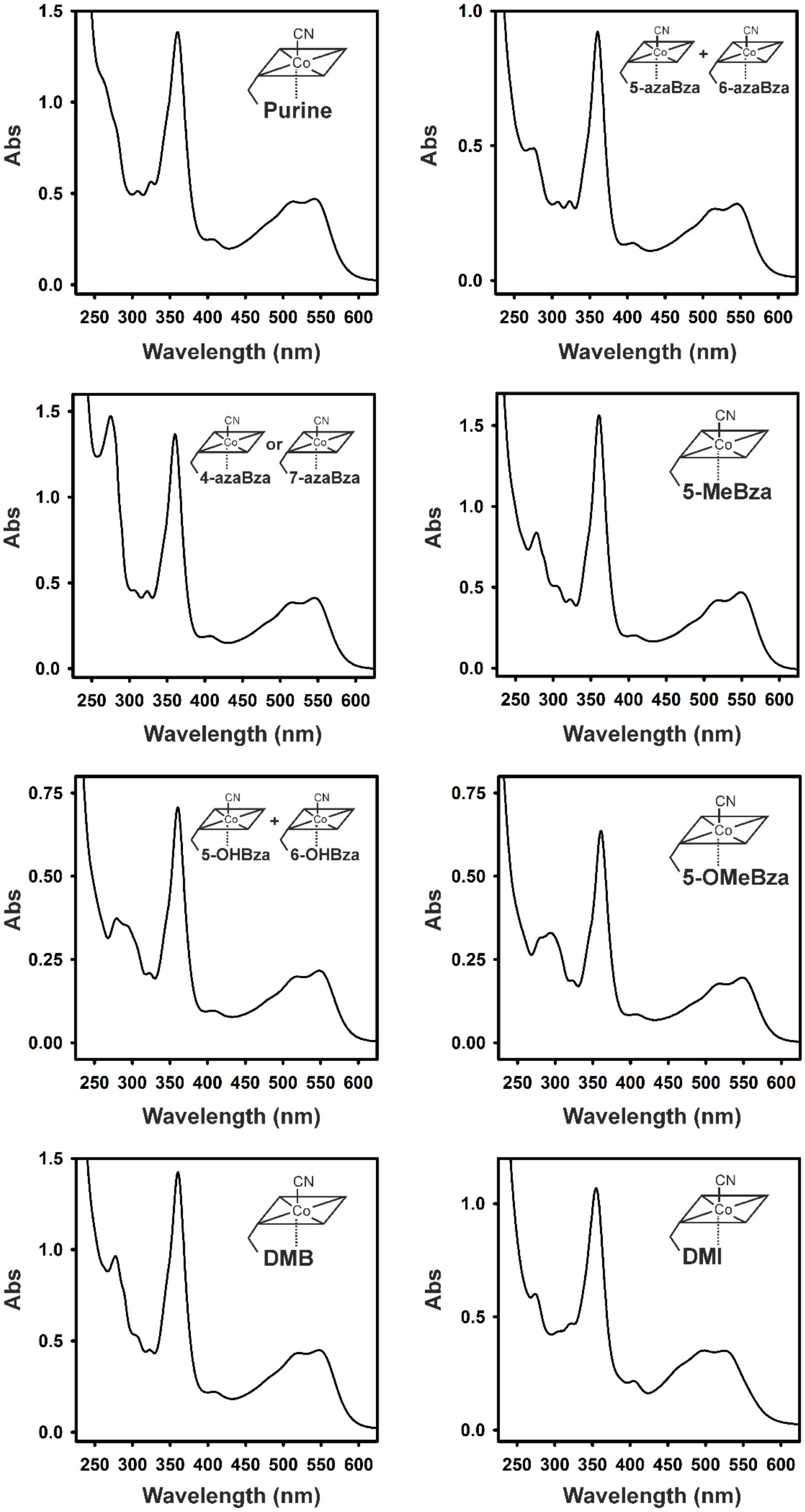

Figure S1: UV/Vis-absorbance spectra of the purified Cbas from *Desulfitobacterium hafniense* strain DCB-2.

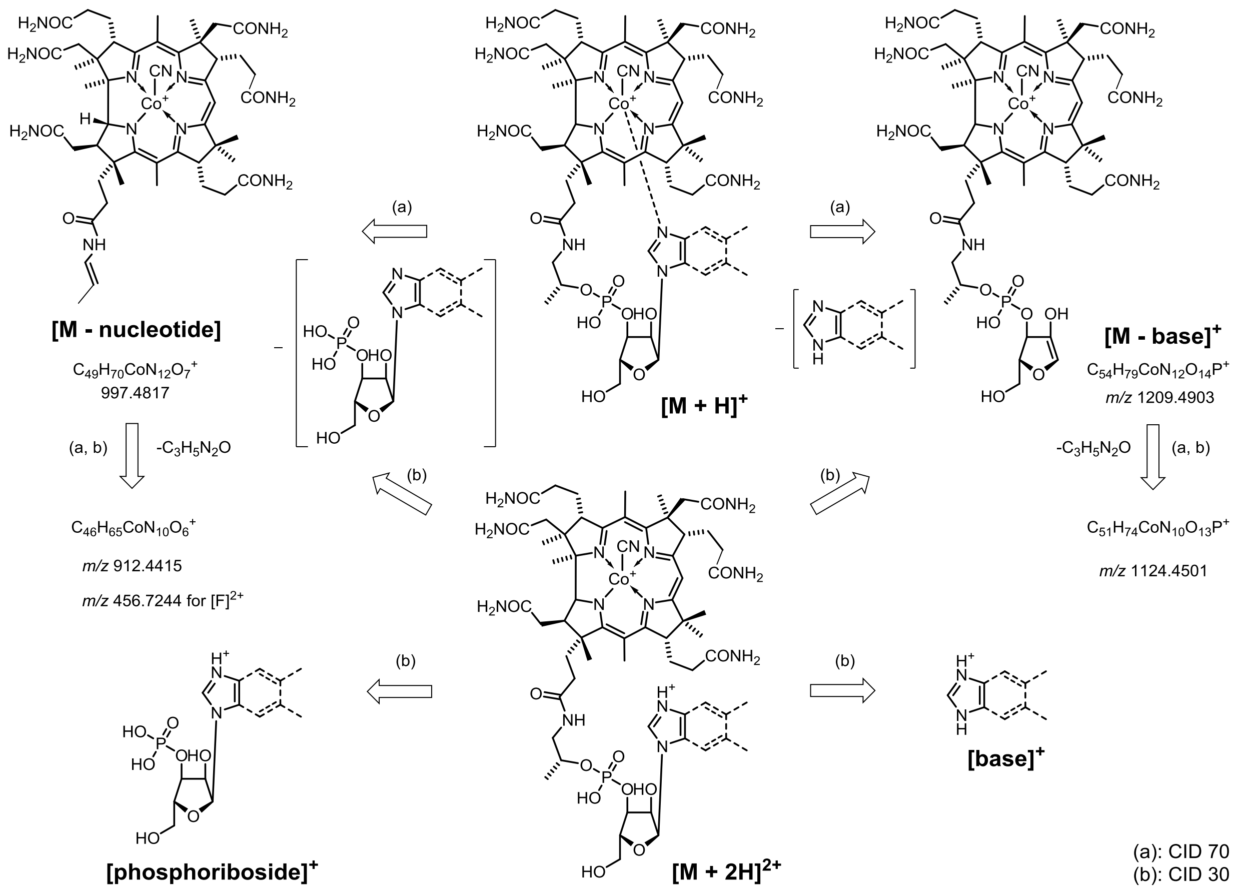

Figure S2. MS/MS fragmentation of cobamides using $[M+H]^+$ and $[M+2H]^{2+}$ precursor ions with a CID energy of 70 and 30, respectively.

Table S1: HPLC-ESI-(+)-HR-MS/MS data of a vitamin B_{12} standard.

	Molecular formula	calc. *m/z*	obs. *m/z*	Δ (ppm)
$[M+H]^+$	$[C_{63}H_{89}CoN_{14}O_{14}P]^+$	1355.5747	1355.5738	0.7
$[M+2H]^{2+}$	$[C_{63}H_{90}CoN_{14}O_{14}P]^{2+}$	678.2910	678.2912	0.3
MS/MS[a]	$[C_{54}H_{79}CoN_{12}O_{14}P]^+$	1209.4903	1209.4893	0.8
MS/MS[a]	$[C_{51}H_{74}CoN_{10}O_{13}P]^+$	1124.4501	1124.4490	1.0
MS/MS[a]	$[C_{49}H_{70}CoN_{12}O_7]^+$	997.4817	997.4805	1.2
MS/MS[a]	$[C_{46}H_{65}CoN_{10}O_6]^+$	912.4415	912.4403	1.3
MS/MS[b]	$[C_{46}H_{66}CoN_{10}O_6]^{2+}$	456.7244	456.7246	0.4
MS/MS[b]	$[C_{14}H_{20}N_2O_7P]^+$	359.1003	359.1003	0.0
MS/MS[b]	$[C_9H_{11}N_2]^+$	147.0917	147.0918	0.7

a: MS/MS of $[M+H]^+$ at CID 70; b: MS/MS of $[M+2H]^{2+}$ at CID 30.

Table S2: HPLC-ESI-(+)-HR-MS/MS data of the purinyl cobamide (signal **1** in Fig. 1) from *D. hafniense* strain DCB-2 supplemented with YE, but without other additives.

	**Molecular formula**	**calc. *m/z***	**obs. *m/z***	**Δ (ppm)**
$[M+H]^+$	$[C_{59}H_{83}CoN_{16}O_{14}P]^+$	1329.5339	1329.5326	1.0
$[M+2H]^{2+}$	$[C_{59}H_{84}CoN_{16}O_{14}P]^{2+}$	665.2726	665.2706	3.0
MS/MS[a]	$[C_{54}H_{79}CoN_{12}O_{14}P]^+$	1209.4903	1209.4890	1.1
MS/MS[a]	$[C_{51}H_{74}CoN_{10}O_{13}P]^+$	1124.4501	1124.4485	1.4
MS/MS[a]	$[C_{49}H_{70}CoN_{12}O_7]^+$	997.4817	997.4800	1.7
MS/MS[a]	$[C_{46}H_{65}CoN_{10}O_6]^+$	912.4415	912.4403	1.3
MS/MS[b]	$[C_{46}H_{66}CoN_{10}O_6]^{2+}$	456.7244	456.7242	0.4
MS/MS[b]	$[C_{10}H_{14}N_4O_7P]^+$	333.0595	333.0596	0.3
MS/MS[b]	$[C_5H_5N_4]^+$	121.0509	121.0504	4.1

a: MS/MS of $[M+H]^+$ at CID 70; b: MS/MS of $[M+2H]^{2+}$ at CID 30.

Table S3: HPLC-ESI-(+)-HR-MS/MS data of the 5-azabenzimidazolyl cobamide (signal **2** in Fig. 1) from *D. hafniense* strain DCB-2 supplemented with YE, but without other additives.

	**Molecular formula**	**calc. *m/z***	**obs. *m/z***	**Δ (ppm)**
$[M+H]^+$	$[C_{60}H_{84}CoN_{15}O_{14}P]^+$	1328.5386	1328.5376	0.8
$[M+2H]^{2+}$	$[C_{60}H_{85}CoN_{15}O_{14}P]^{2+}$	664.7730	664.7729	0.2
MS/MS[a]	$[C_{54}H_{79}CoN_{12}O_{14}P]^+$	1209.4903	1209.4896	0.6
MS/MS[a]	$[C_{51}H_{74}CoN_{10}O_{13}P]^+$	1124.4501	1124.4487	1.2
MS/MS[a]	$[C_{49}H_{70}CoN_{12}O_7]^+$	997.4817	997.4811	0.6
MS/MS[a]	$[C_{46}H_{65}CoN_{10}O_6]^+$	912.4415	912.4405	1.1
MS/MS[b]	$[C_{46}H_{66}CoN_{10}O_6]^{2+}$	456.7244	456.7241	0.7
MS/MS[b]	$[C_{11}H_{15}N_3O_7P]^+$	332.0642	332.0640	0.6
MS/MS[b]	$[C_6H_6N_3]^+$	120.0556	120.0551	4.2

a: MS/MS of $[M+H]^+$ at CID 70; b: MS/MS of $[M+2H]^{2+}$ at CID 30.

Table S4: HPLC-ESI-(+)-HR-MS/MS data of the 5,6-dimethylbenzimidazolyl cobamide from *D. hafniense* strain DCB-2 supplemented with YE and 5,6-dimethylbenzimidazole (DMB).

	**Molecular formula**	**calc. *m/z***	**obs. *m/z***	**Δ (ppm)**
$[M+H]^+$	$[C_{63}H_{89}CoN_{14}O_{14}P]^+$	1355.5747	1355.5745	0.1
$[M+2H]^{2+}$	$[C_{63}H_{90}CoN_{14}O_{14}P]^{2+}$	678.2910	678.2922	1.8
MS/MS[a]	$[C_{54}H_{79}CoN_{12}O_{14}P]^+$	1209.4903	1209.4891	1.0
MS/MS[a]	$[C_{51}H_{74}CoN_{10}O_{13}P]^+$	1124.4501	1124.4493	0.7
MS/MS[a]	$[C_{49}H_{70}CoN_{12}O_7]^+$	997.4817	997.4802	1.5
MS/MS[a]	$[C_{46}H_{65}CoN_{10}O_6]^+$	912.4415	912.4407	0.9
MS/MS[b]	$[C_{46}H_{66}CoN_{10}O_6]^{2+}$	456.7244	456.7246	0.4
MS/MS[b]	$[C_{14}H_{20}N_2O_7P]^+$	359.1003	359.1007	1.1
MS/MS[b]	$[C_9H_{11}N_2]^+$	147.0917	147.0920	2.0

a: MS/MS of $[M+H]^+$ at CID 70; b: MS/MS of $[M+2H]^{2+}$ at CID 30.

Table S5: HPLC-ESI-(+)-HR-MS/MS data of the benzimidazolyl cobamide from *D. hafniense* strain DCB-2 supplemented with YE and benzimidazole (Bza).

	Molecular formula	calc. *m/z*	obs. *m/z*	Δ (ppm)
$[M+H]^+$	$[C_{61}H_{85}CoN_{14}O_{14}P]^+$	1327.5434	1327.5420	1.1
$[M+2H]^{2+}$	$[C_{61}H_{86}CoN_{14}O_{14}P]^{2+}$	664.2753	664.2754	0.2
MS/MS[a]	$[C_{54}H_{79}CoN_{12}O_{14}P]^+$	1209.4903	1209.4886	1.4
MS/MS[a]	$[C_{51}H_{74}CoN_{10}O_{13}P]^+$	1124.4501	1124.4485	1.4
MS/MS[a]	$[C_{49}H_{70}CoN_{12}O_7]^+$	997.4817	997.4801	1.6
MS/MS[a]	$[C_{46}H_{65}CoN_{10}O_6]^+$	912.4415	912.4400	1.6
MS/MS[b]	$[C_{46}H_{66}CoN_{10}O_6]^{2+}$	456.7244	456.7243	0.2
MS/MS[b]	$[C_{12}H_{16}N_2O_7P]^+$	331.0690	331.0682	2.4
MS/MS[b]	$[C_7H_7N_2]^+$	119.0604	119.0600	3.4

a: MS/MS of $[M+H]^+$ at CID 70; b: MS/MS of $[M+2H]^{2+}$ at CID 30.

Table S6: HPLC-ESI-(+)-HR-MS/MS data of the 5-methylbenzimidazolyl cobamide from *D. hafniense* strain DCB-2 supplemented with YE and 5-methylbenzimidazole (5-MeBza).

	Molecular formula	calc. *m/z*	obs. *m/z*	Δ (ppm)
$[M+H]^+$	$[C_{62}H_{87}CoN_{14}O_{14}P]^+$	1341.5590	1341.5573	1.3
$[M+2H]^{2+}$	$[C_{62}H_{88}CoN_{14}O_{14}P]^{2+}$	671.2832	671.2836	0.6
MS/MS[a]	$[C_{54}H_{79}CoN_{12}O_{14}P]^+$	1209.4903	1209.4882	1.7
MS/MS[a]	$[C_{51}H_{74}CoN_{10}O_{13}P]^+$	1124.4501	1124.4478	2.0
MS/MS[a]	$[C_{49}H_{70}CoN_{12}O_7]^+$	997.4817	997.4800	1.7
MS/MS[a]	$[C_{46}H_{65}CoN_{10}O_6]^+$	912.4415	912.4399	1.8
MS/MS[b]	$[C_{46}H_{66}CoN_{10}O_6]^{2+}$	456.7244	456.7251	1.5
MS/MS[b]	$[C_{13}H_{18}N_2O_7P]^+$	345.0846	345.0850	1.2
MS/MS[b]	$[C_8H_9N_2]^+$	133.0760	133.0755	3.8

a: MS/MS of $[M+H]^+$ at CID 70; b: MS/MS of $[M+2H]^{2+}$ at CID 30.

Table S7: HPLC-ESI-(+)-HR-MS/MS data of the 5-methoxybenzimidazolyl cobamide from *D. hafniense* strain DCB-2 supplemented with YE and 5-methoxybenzimidazole (5-OMeBza).

	Molecular formula	calc. *m/z*	obs. *m/z*	Δ (ppm)
$[M+H]^+$	$[C_{62}H_{87}CoN_{14}O_{15}P]^+$	1357.5539	1357.5529	0.7
$[M+2H]^{2+}$	$[C_{62}H_{88}CoN_{14}O_{15}P]^{2+}$	679.2806	679.2811	0.7
MS/MS[a]	$[C_{54}H_{79}CoN_{12}O_{14}P]^+$	1209.4903	1209.4890	1.1
MS/MS[a]	$[C_{51}H_{74}CoN_{10}O_{13}P]^+$	1124.4501	1124.4490	1.0
MS/MS[a]	$[C_{49}H_{70}CoN_{12}O_7]^+$	997.4817	997.4802	1.5
MS/MS[a]	$[C_{46}H_{65}CoN_{10}O_6]^+$	912.4415	912.4400	1.6
MS/MS[b]	$[C_{46}H_{66}CoN_{10}O_6]^{2+}$	456.7244	456.7250	1.3
MS/MS[b]	$[C_{13}H_{18}N_2O_8P]^+$	361.0795	361.0793	0.6
MS/MS[b]	$[C_8H_9N_2O]^+$	149.0709	149.0715	4.0

a: MS/MS of $[M+H]^+$ at CID 70; b: MS/MS of $[M+2H]^{2+}$ at CID 30.

Table S8: HPLC-ESI-(+)-HR-MS/MS data of the 5-/6-hydroxybenzimidazolyl cobamide(s) from *D. hafniense* strain DCB-2 supplemented with YE and 5-hydroxybenzimidazole (5-OHBza)

	Molecular formula	calc. *m/z*	obs. *m/z*	Δ (ppm)
$[M+H]^+$	$[C_{61}H_{85}CoN_{14}O_{15}P]^+$	1343.5383	1343.5375	0.6
$[M+2H]^{2+}$	$[C_{61}H_{86}CoN_{14}O_{15}P]^{2+}$	672.2728	672.2728	0.0
MS/MS[a]	$[C_{54}H_{79}CoN_{12}O_{14}P]^+$	1209.4903	1209.4887	1.3
MS/MS[a]	$[C_{51}H_{74}CoN_{10}O_{13}P]^+$	1124.4501	1124.4484	1.5
MS/MS[a]	$[C_{49}H_{70}CoN_{12}O_7]^+$	997.4817	997.4802	1.5
MS/MS[a]	$[C_{46}H_{65}CoN_{10}O_6]^+$	912.4415	912.4399	1.8
MS/MS[b]	$[C_{46}H_{66}CoN_{10}O_6]^{2+}$	456.7244	456.7243	0.2
MS/MS[b]	$[C_{12}H_{16}N_2O_8P]^+$	347.0639	347.0639	0.0
MS/MS[b]	$[C_7H_7N_2O]^+$	135.0553	135.0551	1.5

a: MS/MS of $[M+H]^+$ at CID 70; b: MS/MS of $[M+2H]^{2+}$ at CID 30.

Table S9: HPLC-ESI-(+)-HR-MS/MS data of the purinyl cobamide (**2**) from *D. hafniense* DCB-2 supplemented with YE and purine

	Molecular formula	calc. *m/z*	obs. *m/z*	Δ (ppm)
$[M+H]^+$	$[C_{59}H_{83}CoN_{16}O_{14}P]^+$	1329.5339	1329.5340	0.1
$[M+2H]^{2+}$	$[C_{59}H_{84}CoN_{16}O_{14}P]^{2+}$	665.2726	665.2723	0.5
MS/MS[a]	$[C_{54}H_{79}CoN_{12}O_{14}P]^+$	1209.4903	1209.4898	0.4
MS/MS[a]	$[C_{51}H_{74}CoN_{10}O_{13}P]^+$	1124.4501	1124.4492	0.8
MS/MS[a]	$[C_{49}H_{70}CoN_{12}O_7]^+$	997.4817	997.4810	0.7
MS/MS[a]	$[C_{46}H_{65}CoN_{10}O_6]^+$	912.4415	912.4407	0.9
MS/MS[b]	$[C_{46}H_{66}CoN_{10}O_6]^{2+}$	456.7244	456.7251	1.5
MS/MS[b]	$[C_{10}H_{14}N_4O_7P]^+$	333.0595	333.0592	0.9
MS/MS[b]	$[C_5H_5N_4]^+$	121.0509	121.0511	1.7

a: MS/MS of $[M+H]^+$ at CID 70; b: MS/MS of $[M+2H]^{2+}$ at CID 30.

Table S10: HPLC-ESI-(+)-HR-MS/MS data of the 5-azabenzimidazolyl cobamide (**2**) from *D. hafniense* DCB-2 supplemented with YE and 5-azabenzimidazole (5-azaBza).

	Molecular formula	calc. *m/z*	obs. *m/z*	Δ (ppm)
$[M+H]^+$	$[C_{60}H_{84}CoN_{15}O_{14}P]^+$	1328.5386	1328.5384	0.2
$[M+2H]^{2+}$	$[C_{60}H_{85}CoN_{15}O_{14}P]^{2+}$	664.7730	664.7752	3.3
MS/MS[a]	$[C_{54}H_{79}CoN_{12}O_{14}P]^+$	1209.4903	1209.4894	0.7
MS/MS[a]	$[C_{51}H_{74}CoN_{10}O_{13}P]^+$	1124.4501	1124.4489	1.1
MS/MS[a]	$[C_{49}H_{70}CoN_{12}O_7]^+$	997.4817	997.4813	0.4
MS/MS[a]	$[C_{46}H_{65}CoN_{10}O_6]^+$	912.4415	912.4412	0.3
MS/MS[a]	$[C_{46}H_{66}CoN_{10}O_6]^{2+}$	456.7244	456.7244	0.0
MS/MS[a]	$[C_{11}H_{15}N_3O_7P]^+$	332.0642	332.0642	0.0
MS/MS[a]	$[C_6H_6N_3]^+$	120.0556	120.0557	0.8

a: MS/MS of $[M+H]^+$ at CID 70; b: MS/MS of $[M+2H]^{2+}$ at CID 30.

Table S11: HPLC-ESI-(+)-HR-MS/MS data of the putative 6-azabenzimidazolyl cobamide (**3**) from *D. hafniense* DCB-2 supplemented with YE and 5-azabenzimidazole (5-azaBza).

	**Molecular formula**	**calc. *m/z***	**obs. *m/z***	**Δ (ppm)**
$[M+H]^+$	$[C_{60}H_{84}CoN_{15}O_{14}P]^+$	1328.5386	1328.5376	0.8
$[M+2H]^{2+}$	$[C_{60}H_{85}CoN_{15}O_{14}P]^{2+}$	664.7730	664.7749	2.9
MS/MS[a]	$[C_{54}H_{79}CoN_{12}O_{14}P]^+$	1209.4903	1209.4886	1.4
MS/MS[a]	$[C_{51}H_{74}CoN_{10}O_{13}P]^+$	1124.4501	1124.4479	2.0
MS/MS[a]	$[C_{49}H_{70}CoN_{12}O_7]^+$	997.4817	997.4812	0.5
MS/MS[a]	$[C_{46}H_{65}CoN_{10}O_6]^+$	912.4415	912.4406	1.0
MS/MS[a]	$[C_{46}H_{66}CoN_{10}O_6]^{2+}$	456.7244	456.7241	0.7
MS/MS[a]	$[C_{11}H_{15}N_3O_7P]^+$	332.0642	332.0641	0.3
MS/MS[a]	$[C_6H_6N_3]^+$	120.0556	120.0559	2.5

a: MS/MS of $[M+H]^+$ at CID 70; b: MS/MS of $[M+2H]^{2+}$ at CID 30.

Table S12: HPLC-ESI-(+)-HR-MS/MS data of the 4-azabenzimidazolyl cobamide (**4**) from *D. hafniense* DCB-2 supplemented with YE and 4-azabenzimidazole (4-azaBza).

	**Molecular formula**	**calc. *m/z***	**obs. *m/z***	**Δ (ppm)**
$[M+H]^+$	$[C_{60}H_{84}CoN_{15}O_{14}P]^+$	1328.5386	1328.5395	0.7
$[M+2H]^{2+}$	$[C_{60}H_{85}CoN_{15}O_{14}P]^{2+}$	664.7730	664.7754	3.6
MS/MS[a]	$[C_{54}H_{79}CoN_{12}O_{14}P]^+$	1209.4903	1209.4899	0.3
MS/MS[a]	$[C_{51}H_{74}CoN_{10}O_{13}P]^+$	1124.4501	1124.4488	1.2
MS/MS[a]	$[C_{49}H_{70}CoN_{12}O_7]^+$	997.4817	997.4810	0.7
MS/MS[a]	$[C_{46}H_{65}CoN_{10}O_6]^+$	912.4415	912.4405	1.1
MS/MS[a]	$[C_{46}H_{66}CoN_{10}O_6]^{2+}$	456.7244	456.7244	0.0
MS/MS[a]	$[C_{11}H_{15}N_3O_7P]^+$	332.0642	332.0638	1.2
MS/MS[a]	$[C_6H_6N_3]^+$	120.0556	120.0553	2.5

a: MS/MS of $[M+H]^+$ at CID 70; b: MS/MS of $[M+2H]^{2+}$ at CID 30.

Table S13: NMR data (700 MHz, D_2O) for the purinyl cobamide (**1**) and the 5-azabenzimidazolyl cobamide (**2**) isolated from *D. hafniense* strain DCB-2.

		**purinyl cobamide (1)**			**5-azabenzimidazolyl cobamide (2)**		
		**^{1}H**	**^{13}C**	**HMBC**	**^{1}H**	**^{13}C**	**HMBC**
Corrinoid	1	-	85.3 *Cq*	-	-	85.3 *Cq*	-
	1a	0.44 *s*	19.8 *CH_3*	1, 2, 19	0.42 *s*	19.62 *CH_3*	1, 2, 19
	2	-	47.1 *Cq*	-	-	47.2 *Cq*	-
	2a	2.39 *s*	42.8 *CH_2*	1, 2, 2a', 2b, 3	2.40 s	42.9 *CH_2*	2, 2a', 2b, 3
	2b	-	175.5 *Cq*	-	-	175.6 *Cq*	-
	2a'	1.40 *s*	16.5 *CH_3*	1, 2, 2a, 3	1.40 s	16.6 *CH_3*	1, 2, 2a, 3
	3	4.20 *m*	56.2 *CH*	1, 2, 2a, 3a, 3b, 4	4.19 *m*	56.2 *CH*	1, 3a, 3b
	3a	1.98 *m*	25.5 *CH_2*	…	1.98 *m*	25.9 *CH_2*	…
	3b	2.53 *m*	34.3 *CH_2*	3c	2.53 *m*	34.6 *CH_2*	3c
	3c	-	177.9 *Cq*	-	-	177.7 *Cq*	-
	4	-	180.7 *Cq*	-	-	180.4 *Cq*	-
	5	-	107.6 *Cq*	-	-	107.5 *Cq*	-
	5a	2.53 *s*	15.2 *CH_3*	4, 5, 6	2.53 *s*	15.4 *CH_3*	4, 5, 6
	6	-	165.5 *Cq*	-	-	165.7 *Cq*	-
	7	-	51.5 *Cq*	-	-	51.3 *Cq*	-
	7a	2.18 *d* 13.7 Hz	42.7 *CH_2*	6, 7, 7a', 7b, 8	2.18 *d* 13.8 Hz	42.3 *CH_2*	6, 7, 7a', 7b, 8
	7a	2.59 *m*	42.7 *CH_2*	7, 7a', 7b, 8	2.59 *m*	42.3 *CH_2*	7, 7a', 7b, 8
	7b	-	174.8 *Cq*	-	-	174.9 *Cq*	-
	7a'	1.88 *s*	18.8 *CH_3*	6, 7, 7a, 8, 8a	1.87 *s*	19.0 *CH_3*	6, 7, 7a, 8
	8	3.54 *dd* 11 Hz, 5 Hz	55.7 *CH*	6, 7, 7a, 8a,	3.51 *dd* 11 Hz, 4.8 Hz	55.7 *CH*	7, 7a, 8a
	8a	2.07 *m* 0.92 *m*	26.3 *CH_2*	…	2.03 *m* 0.91 *m*	26.7 *CH_2*	…
	8b	1.51 *m* 1.95 *m*	31.9 *CH_2*	8c	1.82 *m* 1.32 *m*	32.0 *CH_2*	8c
	8c	-	177.2 *Cq*	-	-	177.1 *Cq*	-
	9	-	174.1 *Cq*	-	-	173.7 *Cq*	-
	10	6.23 *s*	95.4 *CH*	8, 9, 11, 12	6.17 *s*	95.5 *CH*	8, 9, 11, 12
	11	-	177.7 *Cq*	-	-	177.7 *Cq*	-
	12	-	48.5 *Cq*	-	-	48.4 *Cq*	-
	12a	1.48 *s*	18.9 *CH_3*	11, 12, 12a', 13	1.47 *s*	19.0 *CH_3*	11, 12, 12a', 13
	12a'	1.18 *s*	30.8 *CH_3*	11, 12, 12a, 13	1.19 *s*	31.0 *CH_3*	11, 12, 12a, 13
	13	3.39 *d* 9.6 Hz	53.8 *CH*	11, 12, 12a', 13a, 14	3.37 *m*	53.8 *CH*	11, 13a, 13b
	13a	1.95 *m*	28.1 *CH_2*	…	1.96 *m*	28.1 *CH_2*	13c

	13b	2.63 *m*	34.5 *CH₂*	13c	2.63 *m*	34.2 *CH₂*	13c
	13c	-	177.9 *Cq*	-	-	178.*0 Cq*	-
	14	-	166.3 *Cq*	-	-	166.2 *Cq*	-
	15	-	104.5 *Cq*	-	-	104.6 *Cq*	-
	15a	2.60 *s*	14.9 *CH₃*	13, 14, 15,<br>16, 17	2.60 *s*	15.0 *CH₃*	14, 15, 16
	16	-	179.6 *Cq*	-	-	179.4 *Cq*	-
	17	-	59.5 *Cq*	-	-	59.5 *Cq*	-
	17a	1.82 *m*<br>2.67 *m*	32.3 *CH₂*	…	1.83 *m*<br>2.66 *m*	32.3 *CH₂*	…
	17b	2.09 *m*<br>2.55 *m*	32.5 *CH₂*	17c	2.10 *m*<br>2.54 *m*	32.8 *CH₂*	17c
	17c	-	177.5 *Cq*	-	-	178.0 *Cq*	-
	17a’	1.39 *s*	15.9 *CH₃*	16, 17,<br>17a, 18	1.39 *s*	16.0 *CH₃*	16, 17,<br>17a, 18
	18	2.80 *m*	38.8 *CH*	17, 18a	2.79 *m*	38.9 *CH*	17a’, 19
	18a	2.69 *m*<br>2.76 *m*	31.5 *CH₂*	17, 18,<br>18b	2.69 *m*<br>2.75 *m*	31.5 *CH₂*	17, 17a’,<br>18, 18b,19
	18b	-	175.3 *Cq*	-	-	175.2 *Cq*	-
	19	4.13 *d*<br>11.0 Hz	75.2 *CH*	1, 1a’, 16,<br>18	4.12 *d*<br>10.7 Hz	75.2 *CH*	…

C3-linker	1’	2.93 *m*<br>3.59 *m*	45.3 *CH₂*	…	2.92 *m*<br>3.61 *m*	45.4 *CH₂*	…
	2’	4.30 *m*	72.9 *CH*	…	4.30 *m*	72.9 *CH*	…
	3’	1.25 *d*<br>6.1 Hz	18.8 *CH₃*	1’, 2’	1.25 *d*<br>6.0 Hz	19.0 *CH₃*	1’, 2’

RIBOSE	1’’	6.61 *d*<br>3 Hz	86.5 *CH*	…	6.47 *d*	87.5 *CH*	2’’, 2’’’
	2’’	4.31 *m*	69.0 *CH*	…	4.31 *m*	67.4 *CH*	…
	3’’	4.74 *m*	72.9 *CH*	…	4.72 *m*	73.0 *CH*	…
	4’’	4.10 *m*	82.6 *CH*	…	4.09 *m*	82.5 *CH*	…
	5’’	3.74 *dd*<br>13.0, 3.6<br>Hz<br>3.92 *d*<br>12.3 Hz	60.2 *CH₂*	…	3.76 *dd*<br><br>3.93 *dd*	60.4 *CH₂*	3’’<br>3’’

LIGAND	2’’’	7.41 *s*	146.5 *CH*	3a’’’, 7a’’’	7.30 *s*	144.8.2<br>*CH*	3a’’’, 7a’’’
	3a’’’	-	129.5 *Cq*	-	-	135.5 *Cq*	-
	4’’’	-	-	-	8.00 *s*	138.2 *CH*	3a’’’, 7a’’’,<br>6’’’
	5’’’	8.19 *s*	145.3 *CH*	3a’’’, 7a’’’,<br>4’’’	-	-	-
	6’’’	-	-	-	8.37 *d*<br>5.0 Hz	143.0 *CH*	7’’’, 7a’’’
	7’’’	8.95 *s*	153.5 *CH*	6’’’	7.61 *d*<br>5.2 Hz	107.9 *CH*	3a’’’
	7a’’’	-	149.5 *Cq*	-	-	137.3 *Cq*	-

Obtained from 700 MHz ^{1}H PURGE NMR, PRESAT-*dqf*-COSY, HSQC, HMBC, and PRESAT-ROESY spectra; Referenced to water at δ_H 4.79 ppm and acetate at δ_H 1.91

Figure S3: Section of the HMBC spectrum of the purinyl cobamide (**1**) isolated from *D. hafniense* strain DCB-2 showing H,C-correlations in the purine unit.

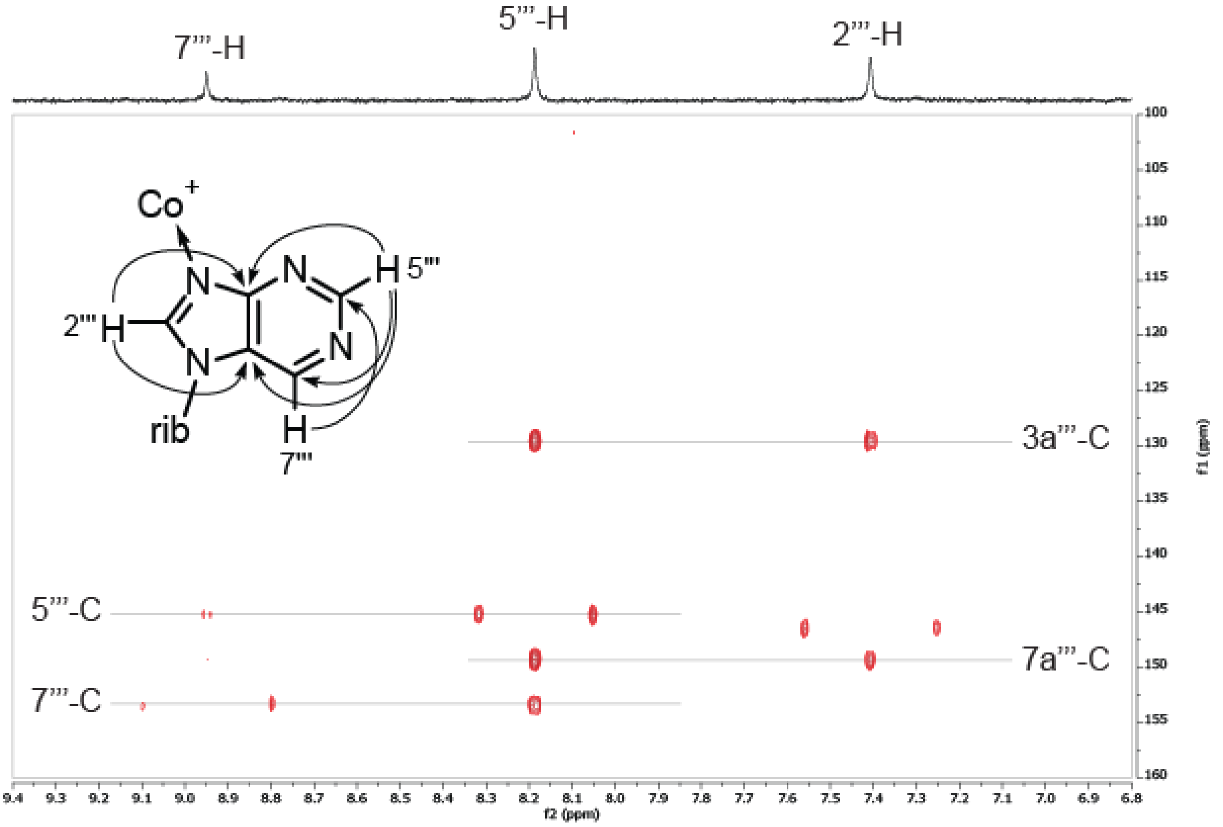

Figure S4: Section of the HMBC spectrum of the 5-azabenzimidazolyl cobamide (**2**) isolated from *D. hafniense* strain DCB-2 showing H,C-correlations in the 5-azabenzimidazole unit.

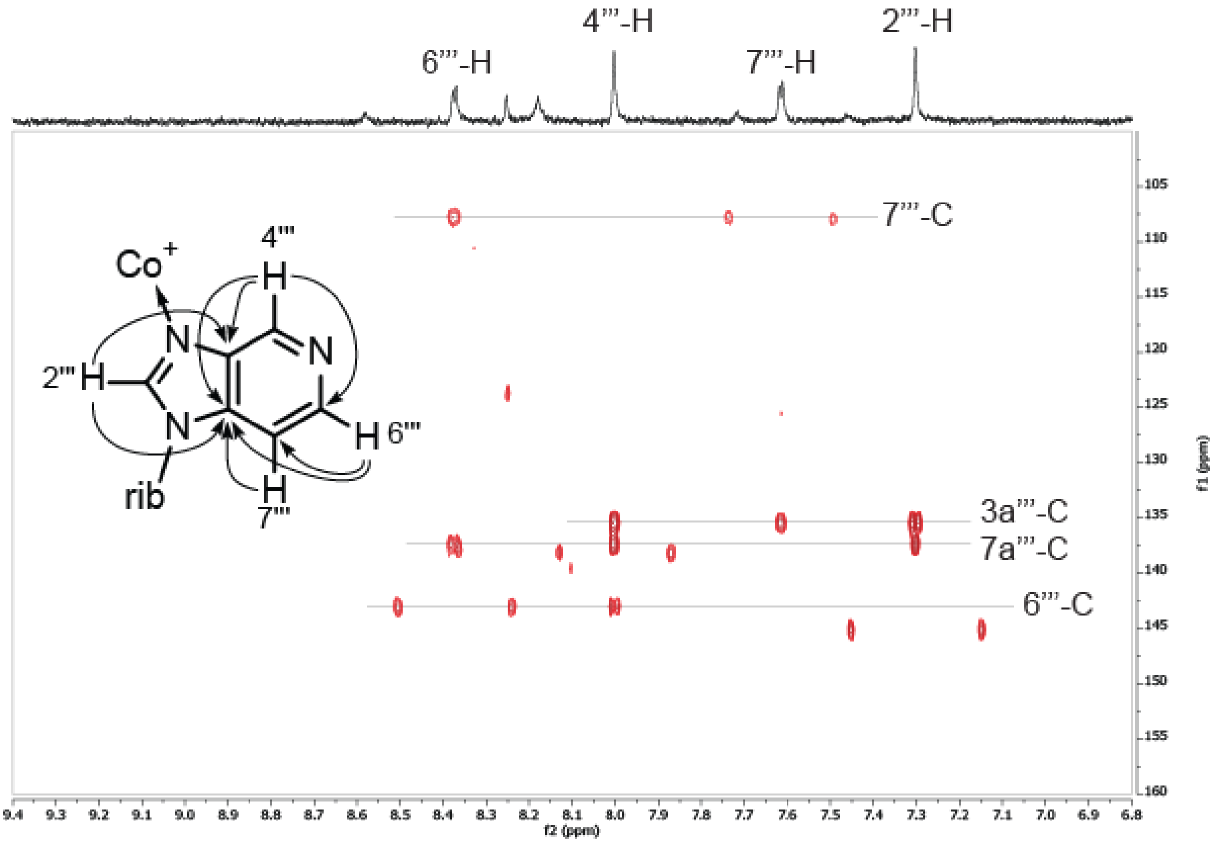

purinyl cobamide (**1**) 5-azabenzimidazolyl cobamide (**2**)

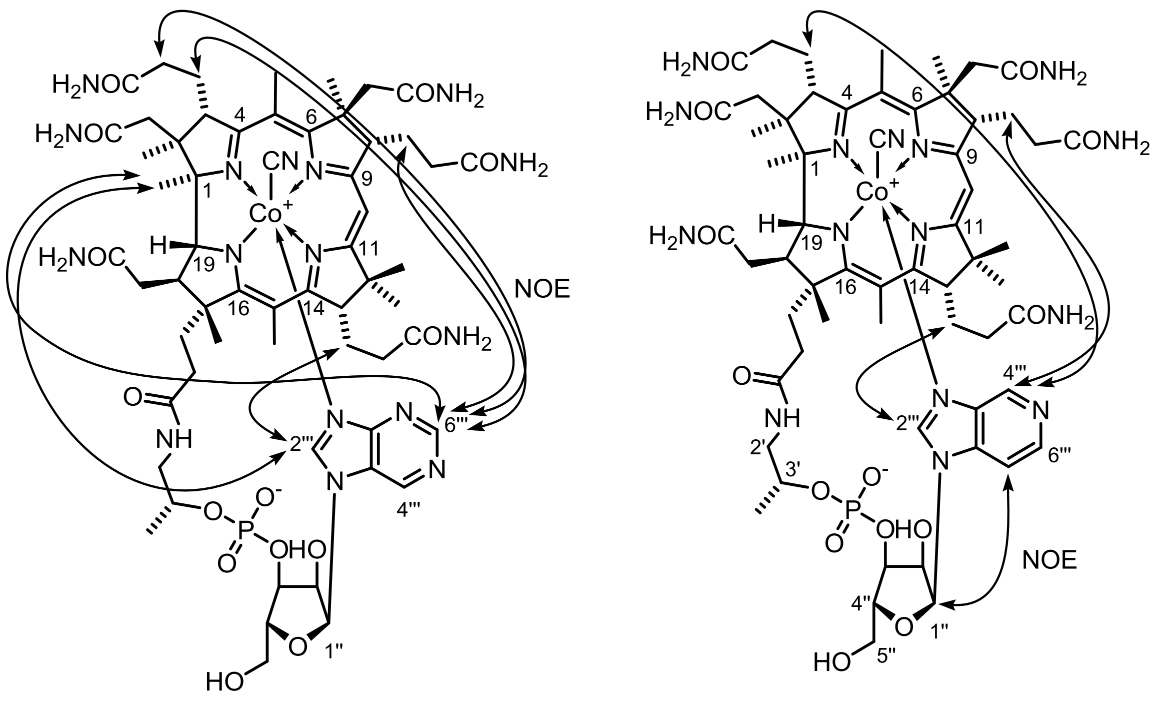

Figure S5: Orientation of the heteroaromatic ligands as deduced from analysis of NOE-correlations observed in the 700 MHz ROESY spectrum.

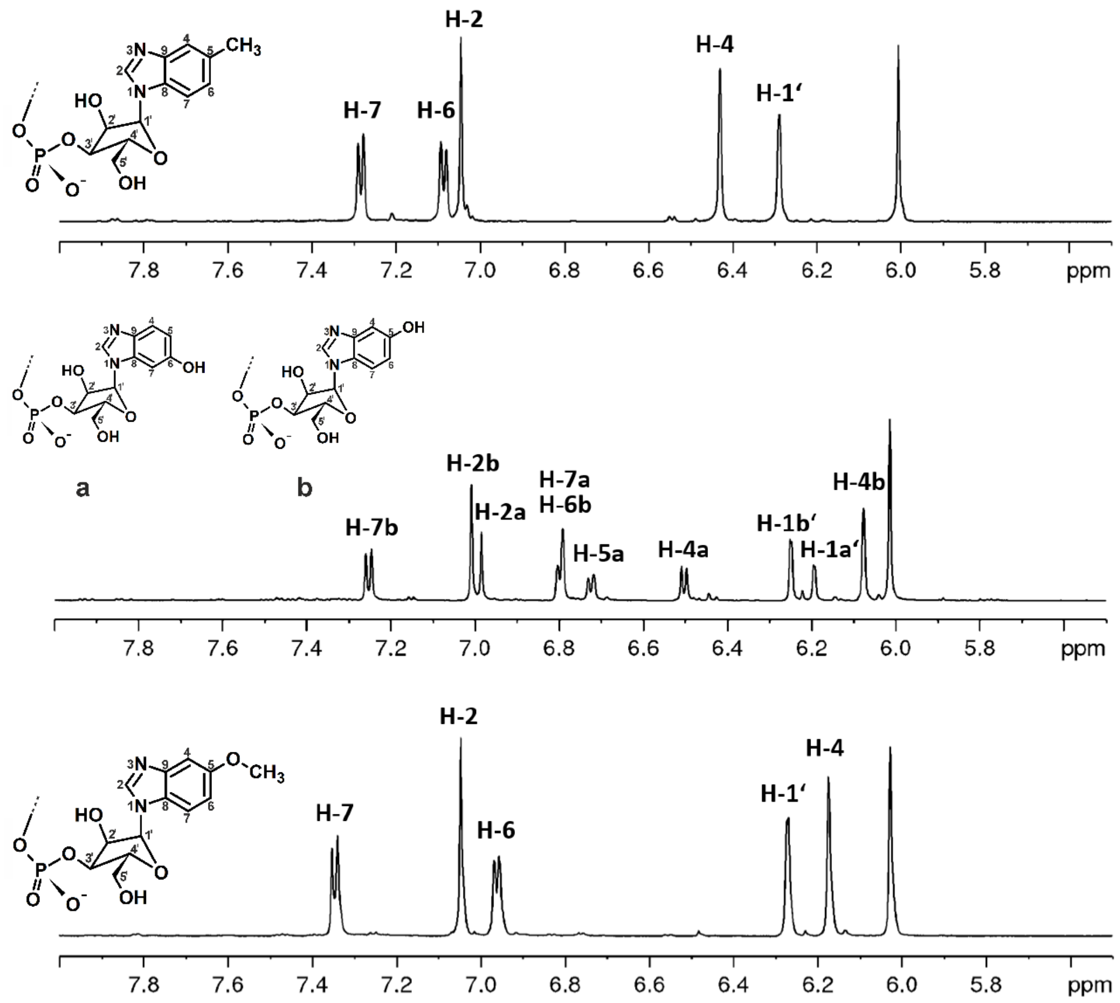

Figure S6. Low field range of ^{1}H-NMR spectra of 5-MeBza-Cba, 6-OHBza-Cba and 5-OHBza-Cba, and 5-OMeBza-Cba. The depicted sections show the signals for the respective benzimidazolyl moieties and the signal for the anomeric position of the α-ribosyl unit.

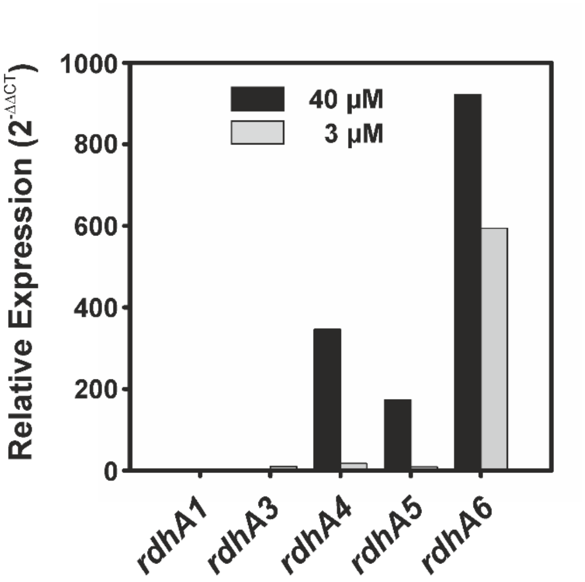

Figure S7. Relative transcript levels of the *rdhA1*, *rdhA3*, *rdhA4*, *rdhA5*, and *rdhA6* genes in two cultures of *D. hafniense* strain DCB-2. Cells were cultivated with pyruvate. When the cultures reached an OD_{578} = 0.2, 3-chloro-4-hydroxy-phenylacetate (ClOHPA; 100 µM) was added. The concentration of ClOHPA in the cultures before the harvest of the cells (OD_{578} = 0.3) is given in the figure.

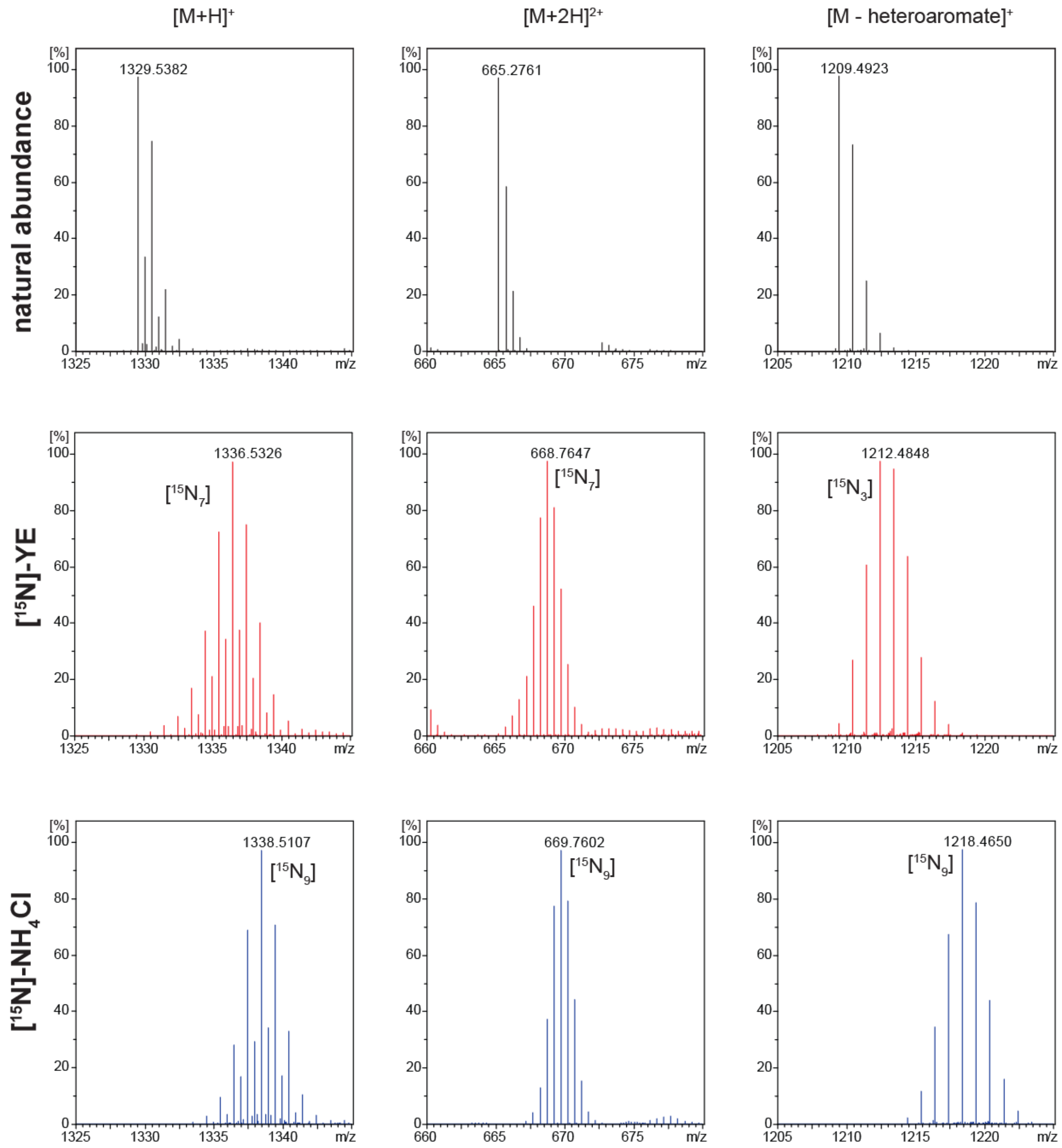

Figure S8A: HR-MS/MS analysis of the purinyl cobamide (**1**) from *D. hafniense* strain DCB-2 supplemented with [15N]-enriched yeast extract or [15N]-enriched NH_4Cl.

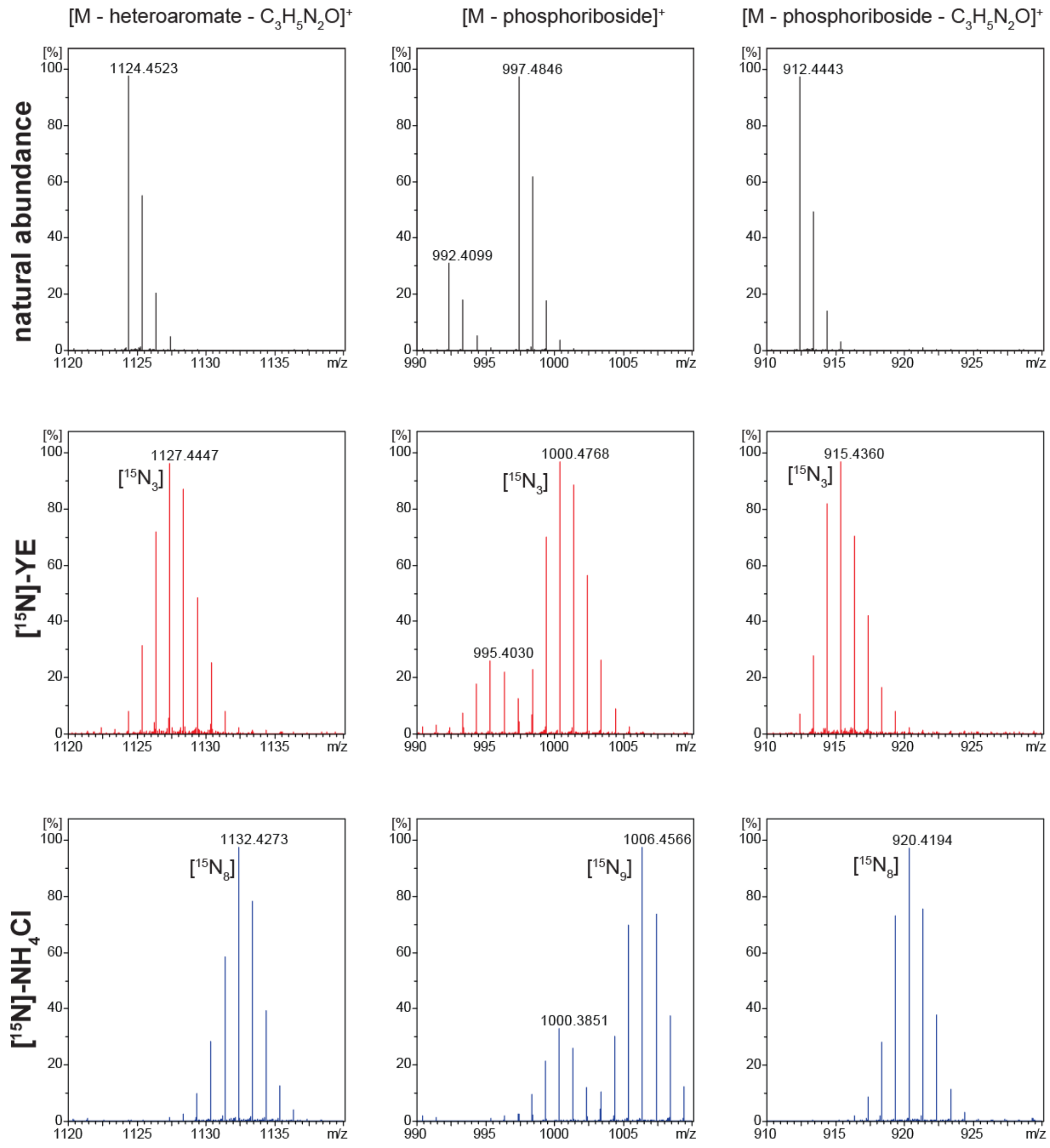

Figure S8B: HR-MS/MS analysis of the purinyl cobamide (**1**) from *D. hafniense* strain DCB-2 supplemented with [^{15}N]-enriched yeast extract or [^{15}N]-enriched NH_4Cl.

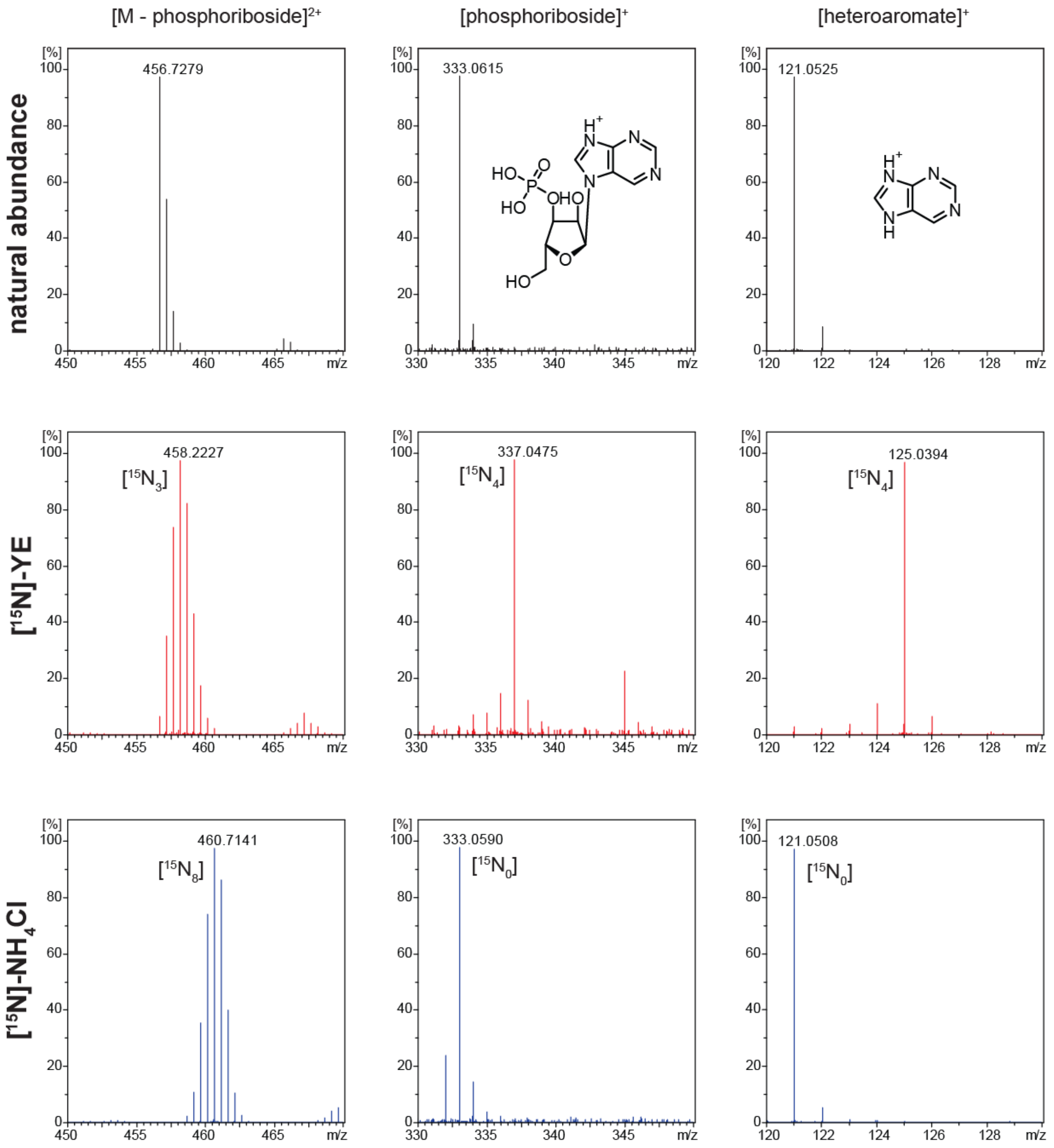

Figure S8C: HR-MS/MS analysis of the purinyl cobamide (**1**) from *D. hafniense* strain DCB-2 supplemented with [^{15}N]-enriched yeast extract or [^{15}N]-enriched NH_4Cl.

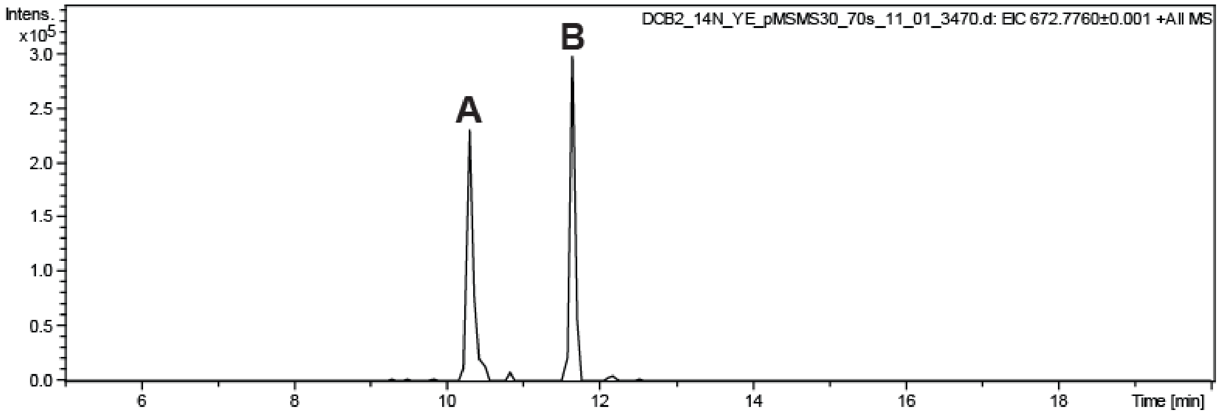

Figure S9: HPLC-ESI-(+)-HR-MS chromatogram of $[M+2H]^{2+}$ signals corresponding to putative adeninyl cobamides from *D. hafniense* strain DCB-2 supplemented with the ^{15}N-enriched YE.

Table S14: HPLC-ESI-(+)-HR-MS/MS data of putative adeninyl cobamides from *D. hafniense* strain DCB-2 supplemented with the ^{15}N-enriched YE.

A	Molecular formula	calc. *m/z*	obs. *m/z*	Δ (ppm)
$[M+H]^+$	$[C_{59}H_{84}CoN_{17}O_{14}P]^+$	1344.5448	1344.5434	1.1
$[M+2H]^{2+}$	$[C_{59}H_{85}CoN_{17}O_{14}P]^{2+}$	672.7760	672.7766	0.9
MS/MS[b]	$[C_{54}H_{79}CoN_{12}O_{14}P]^+$	1209.4903	1209.4880	1.9
MS/MS[b]	$[C_{51}H_{74}CoN_{10}O_{13}P]^+$	1124.4501	1124.4487	1.2
MS/MS[b]	$[C_{49}H_{70}CoN_{12}O_7]^+$	997.4817	997.4820	0.3
MS/MS[b]	$[C_{46}H_{65}CoN_{10}O_6]^+$	912.4415	912.4417	0.2
MS/MS[b]	$[C_{46}H_{66}CoN_{10}O_6]^{2+}$	456.7244	456.7243	0.2
MS/MS[b]	$[C_{10}H_{15}N_5O_7P]^+$	348.0704	348.0704	0.2
MS/MS[b]	$[C_5H_6N_5]^+$	136.0618	136.0615	1.8

B	Molecular formula	calc. *m/z*	obs. *m/z*	Δ (ppm)
$[M+H]^+$	$[C_{59}H_{84}CoN_{17}O_{14}P]^+$	1344.5448	1344.5421	2.0
$[M+2H]^{2+}$	$[C_{59}H_{85}CoN_{17}O_{14}P]^{2+}$	672.7760	672.7762	0.3
MS/MS[b]	$[C_{54}H_{79}CoN_{12}O_{14}P]^+$	1209.4903	1209.4875	2.3
MS/MS[b]	$[C_{51}H_{74}CoN_{10}O_{13}P]^+$	1124.4501	1124.4494	0.6
MS/MS[b]	$[C_{49}H_{70}CoN_{12}O_7]^+$	997.4817	997.4797	2.0
MS/MS[b]	$[C_{46}H_{65}CoN_{10}O_6]^+$	912.4415	912.4401	1.5
MS/MS[b]	$[C_{46}H_{66}CoN_{10}O_6]^{2+}$	456.7244	456.7238	1.3
MS/MS[b]	$[C_{10}H_{15}N_5O_7P]^+$	348.0704	348.0703	0.0
MS/MS[b]	$[C_5H_6N_5]^+$	136.0618	136.0620	1.7

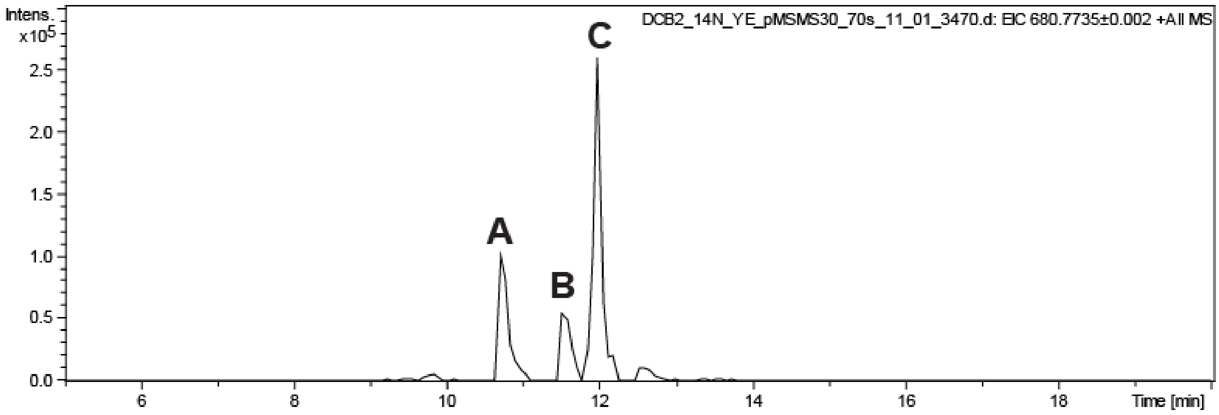

Figure S10: HPLC-ESI-(+)-HR-MS chromatogram of $[M+2H]^{2+}$ signals corresponding to putative guaninyl cobamides from *D. hafniense* strain DCB-2 supplemented with the ^{15}N-enriched YE.

Table S15: HPLC-ESI-(+)-HR-MS/MS data of putative guaninyl cobamides from *D. hafniense* strain DCB-2 supplemented with the ^{15}N-enriched YE.

A	Molecular formula	calc. *m/z*	obs. *m/z*	Δ (ppm)
$[M+H]^+$	$[C_{59}H_{84}CoN_{17}O_{15}P]^+$	1360.5397	1360.5431	2.5
$[M+2H]^{2+}$	$[C_{59}H_{85}CoN_{17}O_{15}P]^{2+}$	680.7735	680.7725	1.4
MS/MS[b]	$[C_{54}H_{79}CoN_{12}O_{14}P]^+$	1209.4903	1209.4896	0.6
MS/MS[b]	$[C_{51}H_{74}CoN_{10}O_{13}P]^+$	1124.4501	1124.4479	2.0
MS/MS[b]	$[C_{49}H_{70}CoN_{12}O_7]^+$	997.4817	997.4811	0.6
MS/MS[b]	$[C_{46}H_{65}CoN_{10}O_6]^+$	912.4415	912.4411	0.4
MS/MS[b]	$[C_{46}H_{66}CoN_{10}O_6]^{2+}$	456.7244	456.7256	2.6
MS/MS[b]	$[C_{10}H_{15}N_5O_8P]^+$	364.0653	364.0641	3.3
MS/MS[b]	$[C_5H_6N_5O]^+$	152.0567	152.0564	1.6

B	Molecular formula	calc. *m/z*	obs. *m/z*	Δ (ppm)
$[M+H]^+$	$[C_{59}H_{84}CoN_{17}O_{15}P]^+$	1360.5397	1360.5411	1.1
$[M+2H]^{2+}$	$[C_{59}H_{85}CoN_{17}O_{15}P]^{2+}$	680.7735	680.7743	1.1
MS/MS[b]	$[C_{54}H_{79}CoN_{12}O_{14}P]^+$	1209.4903	nd	-
MS/MS[b]	$[C_{51}H_{74}CoN_{10}O_{13}P]^+$	1124.4501	nd	-
MS/MS[b]	$[C_{49}H_{70}CoN_{12}O_7]^+$	997.4817	nd	-
MS/MS[b]	$[C_{46}H_{65}CoN_{10}O_6]^+$	912.4415	nd	-
MS/MS[b]	$[C_{46}H_{66}CoN_{10}O_6]^{2+}$	456.7244	nd	-
MS/MS[b]	$[C_{10}H_{15}N_5O_8P]^+$	364.0653	nd	-
MS/MS[b]	$[C_5H_6N_5O]^+$	152.0567	nd	-

C	Molecular formula	calc. *m/z*	obs. *m/z*	Δ (ppm)
$[M+H]^+$	$[C_{59}H_{84}CoN_{17}O_{15}P]^+$	1360.5397	1360.5413	1.2
$[M+2H]^{2+}$	$[C_{59}H_{85}CoN_{17}O_{15}P]^{2+}$	680.7735	680.7745	1.4
MS/MS[b]	$[C_{54}H_{79}CoN_{12}O_{14}P]^+$	1209.4903	1209.4885	1.5
MS/MS[b]	$[C_{51}H_{74}CoN_{10}O_{13}P]^+$	1124.4501	1124.4466	3.1
MS/MS[b]	$[C_{49}H_{70}CoN_{12}O_7]^+$	997.4817	997.4805	1.2
MS/MS[b]	$[C_{46}H_{65}CoN_{10}O_6]^+$	912.4415	912.4396	2.1
MS/MS[b]	$[C_{46}H_{66}CoN_{10}O_6]^{2+}$	456.7244	456.7251	1.5
MS/MS[b]	$[C_{10}H_{15}N_5O_8P]^+$	364.0653	364.0640	3.4
MS/MS[b]	$[C_5H_6N_5O]^+$	152.0567	152.0569	1.6

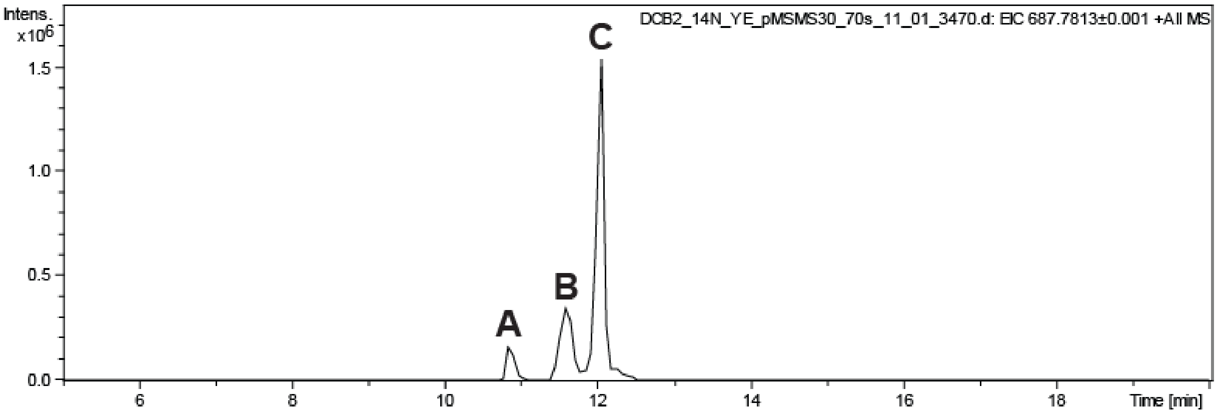

Figure S11: HPLC-ESI-(+)-HR-MS chromatogram of $[M+2H]^{2+}$ signals corresponding to putative methylguaninyl cobamides from *D. hafniense* strain DCB-2 supplemented with the ^{15}N-enriched YE.

Table S16: HPLC-ESI-(+)-HR-MS/MS data of putative methylguaninyl cobamides from *D. hafniense* strain DCB-2 supplemented with the ^{15}N-enriched YE.

**A**	**Molecular formula**	**calc. *m/z***	**obs. *m/z***	**Δ (ppm)**
$[M+H]^+$	$[C_{60}H_{86}CoN_{17}O_{15}P]^+$	1374.5553	1374.5549	0.3
$[M+2H]^{2+}$	$[C_{60}H_{87}CoN_{17}O_{15}P]^{2+}$	687.7813	687.7821	1.2
MS/MS^b	$[C_{54}H_{79}CoN_{12}O_{14}P]^+$	1209.4903	1209.4903	0.0
MS/MS^b	$[C_{51}H_{74}CoN_{10}O_{13}P]^+$	1124.4501	1124.4508	0.6
MS/MS^b	$[C_{49}H_{70}CoN_{12}O_7]^+$	997.4817	997.4810	0.7
MS/MS^b	$[C_{46}H_{65}CoN_{10}O_6]^+$	912.4415	912.4417	0.2
MS/MS^b	$[C_{46}H_{66}CoN_{10}O_6]^{2+}$	456.7244	456.7243	0.2
MS/MS^b	$[C_{11}H_{17}N_5O_8P]^+$	378.0809	378.0816	1.7
MS/MS^b	$[C_6H_8N_5O]^+$	166.0723	166.0724	0.3

**B**	**Molecular formula**	**calc. *m/z***	**obs. *m/z***	**Δ (ppm)**
$[M+H]^+$	$[C_{60}H_{86}CoN_{17}O_{15}P]^+$	1374.5553	1374.5531	1.7
$[M+2H]^{2+}$	$[C_{60}H_{87}CoN_{17}O_{15}P]^{2+}$	687.7813	687.7819	0.9
MS/MS^b	$[C_{54}H_{79}CoN_{12}O_{14}P]^+$	1209.4903	1209.4888	1.2
MS/MS^b	$[C_{51}H_{74}CoN_{10}O_{13}P]^+$	1124.4501	1124.4479	2.0
MS/MS^b	$[C_{49}H_{70}CoN_{12}O_7]^+$	997.4817	997.4803	1.4
MS/MS^b	$[C_{46}H_{65}CoN_{10}O_6]^+$	912.4415	912.4406	1.0
MS/MS^b	$[C_{46}H_{66}CoN_{10}O_6]^{2+}$	456.7244	456.7255	2.4
MS/MS^b	$[C_{11}H_{17}N_5O_8P]^+$	378.0809	378.0824	2.3
MS/MS^b	$[C_6H_8N_5O]^+$	166.0723	166.0724	0.3

C	Molecular formula	calc. *m/z*	obs. *m/z*	Δ (ppm)
$[M+H]^+$	$[C_{60}H_{86}CoN_{17}O_{15}P]^+$	1374.5553	1374.5544	2.8
$[M+2H]^{2+}$	$[C_{60}H_{87}CoN_{17}O_{15}P]^{2+}$	687.7813	687.7813	0.0
MS/MS[b]	$[C_{54}H_{79}CoN_{12}O_{14}P]^+$	1209.4903	1209.4891	1.0
MS/MS[b]	$[C_{51}H_{74}CoN_{10}O_{13}P]^+$	1124.4501	1124.4481	1.8
MS/MS[b]	$[C_{49}H_{70}CoN_{12}O_7]^+$	997.4817	997.4804	1.3
MS/MS[b]	$[C_{46}H_{65}CoN_{10}O_6]^+$	912.4415	912.4400	1.6
MS/MS[b]	$[C_{46}H_{66}CoN_{10}O_6]^{2+}$	456.7244	456.7244	0.0
MS/MS[b]	$[C_{11}H_{17}N_5O_8P]^+$	378.0809	378.0816	0.1
MS/MS[b]	$[C_6H_8N_5O]^+$	166.0723	166.0725	0.8

a: MS/MS of $[M+H]^+$ at CID 70; b: MS/MS of $[M+2H]^{2+}$ at CID 30.

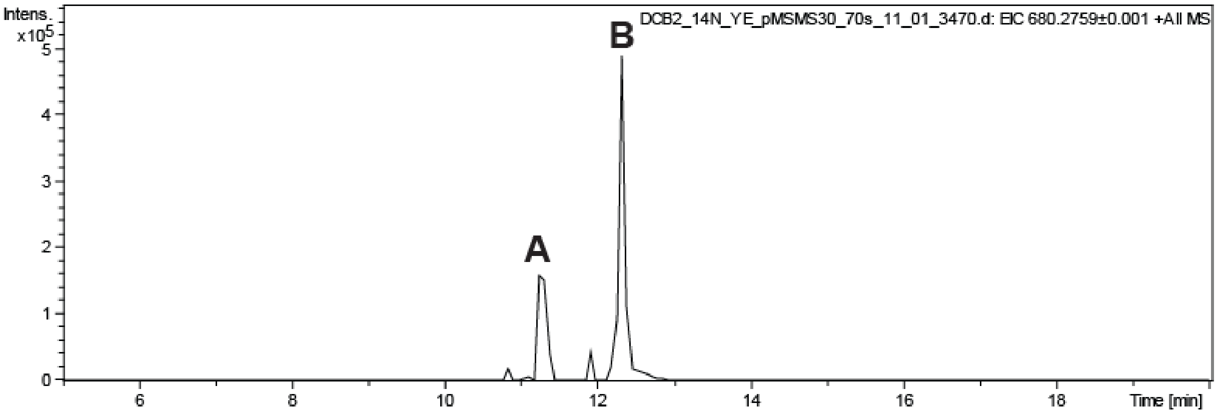

Figure S12: HPLC-ESI-(+)-HR-MS chromatogram of $[M+2H]^{2+}$ signals corresponding to putative methylhypoxanthinyl cobamides from *D. hafniense* strain DCB-2 supplemented with the ^{15}N-enriched YE.

Table S17: HPLC-ESI-(+)-HR-MS/MS data of putative methylhypoxanthinyl cobamides from *D. hafniense* strain DCB-2 supplemented with the ^{15}N-enriched YE.

A	Molecular formula	calc. *m/z*	obs. *m/z*	Δ (ppm)
$[M+H]^+$	$[C_{60}H_{85}CoN_{16}O_{15}P]^+$	1359.5444	1359.5419	1.8
$[M+2H]^{2+}$	$[C_{60}H_{86}CoN_{16}O_{15}P]^{2+}$	680.2759	680.2766	1.1
MS/MS^b	$[C_{54}H_{79}CoN_{12}O_{14}P]^+$	1209.4903	1209.4896	0.6
MS/MS^b	$[C_{51}H_{74}CoN_{10}O_{13}P]^+$	1124.4501	1124.4479	2.0
MS/MS^b	$[C_{49}H_{70}CoN_{12}O_7]^+$	997.4817	997.4811	0.6
MS/MS^b	$[C_{46}H_{65}CoN_{10}O_6]^+$	912.4415	912.4411	0.4
MS/MS^b	$[C_{46}H_{66}CoN_{10}O_6]^{2+}$	456.7244	456.7256	2.6
MS/MS^b	$[C_{11}H_{16}N_4O_8P]^+$	363.0700	363.0697	0.9
MS/MS^b	$[C_6H_7N_4O]^+$	151.0614	151.0615	0.6

B	Molecular formula	calc. *m/z*	obs. *m/z*	Δ (ppm)
$[M+H]^+$	$[C_{60}H_{85}CoN_{16}O_{15}P]^+$	1359.5444	1359.5401	3.2
$[M+2H]^{2+}$	$[C_{60}H_{86}CoN_{16}O_{15}P]^{2+}$	680.2759	680.2761	0.3
MS/MS^b	$[C_{54}H_{79}CoN_{12}O_{14}P]^+$	1209.4903	1209.4874	2.4
MS/MS^b	$[C_{51}H_{74}CoN_{10}O_{13}P]^+$	1124.4501	1124.4468	2.9
MS/MS^b	$[C_{49}H_{70}CoN_{12}O_7]^+$	997.4817	997.4796	2.1
MS/MS^b	$[C_{46}H_{65}CoN_{10}O_6]^+$	912.4415	912.4390	2.7
MS/MS^b	$[C_{46}H_{66}CoN_{10}O_6]^{2+}$	456.7244	456.7242	0.4
MS/MS^b	$[C_{11}H_{16}N_4O_8P]^+$	363.0700	363.0695	1.5
MS/MS^b	$[C_6H_7N_4O]^+$	151.0614	151.0613	1.2

a: MS/MS of $[M+H]^+$ at CID 70; b: MS/MS of $[M+2H]^{2+}$ at CID 30.

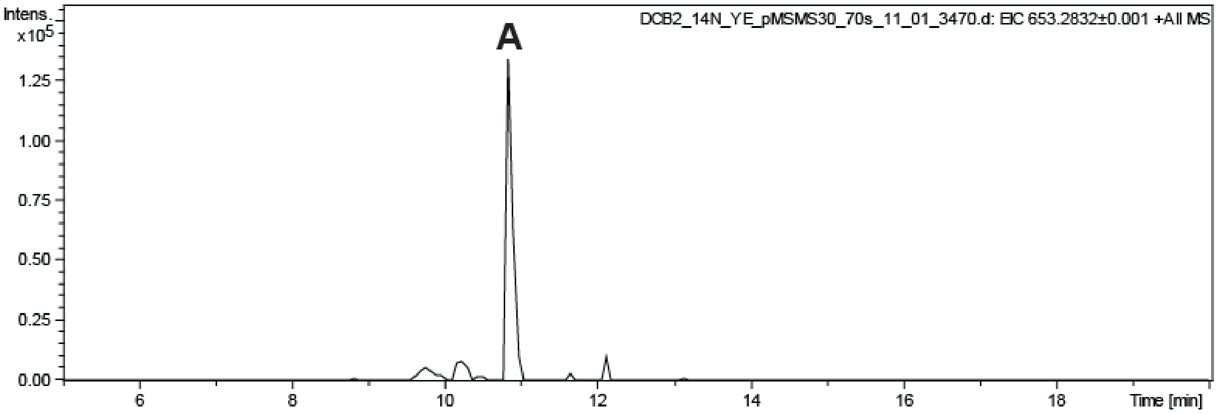

Figure S13: HPLC-ESI-(+)-HR-MS chromatogram of $[M+2H]^{2+}$ signals and HPLC-ESI-(+)-HR-MS/MS data of dimethylimidazolyl cobamide from *D. hafniense* strain DCB-2 supplemented with the ^{15}N-enriched YE.

Table S18: HPLC-ESI-(+)-HR-MS data of putative dimethylimidazolyl cobamide from *D. hafniense* strain DCB-2 supplemented with the ^{15}N-enriched YE.

A	Molecular formula	calc. *m/z*	obs. *m/z*	Δ (ppm)
$[M+H]^+$	$[C_{59}H_{87}CoN_{14}O_{14}P]^+$	1305.5590	1305.5579	0.9
$[M+2H]^{2+}$	$[C_{59}H_{88}CoN_{14}O_{14}P]^{2+}$	653.2832	653.2838	1.1
MS/MS[b]	$[C_{54}H_{79}CoN_{12}O_{14}P]^+$	1209.4903	1209.4886	1.4
MS/MS[b]	$[C_{51}H_{74}CoN_{10}O_{13}P]^+$	1124.4501	1124.4510	0.8
MS/MS[b]	$[C_{49}H_{70}CoN_{12}O_7]^+$	997.4817	997.4807	1.0
MS/MS[b]	$[C_{46}H_{65}CoN_{10}O_6]^+$	912.4415	912.4373	4.6
MS/MS[b]	$[C_{46}H_{66}CoN_{10}O_6]^{2+}$	456.7244	456.7225	4.2
MS/MS[b]	$[C_{10}H_{18}N_2O_7P]^+$	309.0846	309.0842	1.2
MS/MS[b]	$[C_5H_9N_2]^+$	97.0760	nd	-

a: MS/MS of $[M+H]^+$ at CID 70; b: MS/MS of $[M+2H]^{2+}$ at CID 30.

Table S19: HPLC-ESI-(+)-HR-MS/MS data of the 4,5-dimethylimidazolyl cobamide (signal **5** in Fig. 4) from *D. hafniense* strain DCB-2 supplemented with 4,5-dimethylimidazole (DMI).

	Molecular formula	calc. *m/z*	obs. *m/z*	Δ (ppm)
$[M+H]^+$	$[C_{59}H_{87}CoN_{14}O_{14}P]^+$	1305.5590	1305.5605	1.1
$[M+2H]^{2+}$	$[C_{59}H_{88}CoN_{14}O_{14}P]^{2+}$	653.2832	653.2858	4.0
MS/MS[a]	$[C_{54}H_{79}CoN_{12}O_{14}P]^+$	1209.4903	1209.4909	0.5
MS/MS[a]	$[C_{51}H_{74}CoN_{10}O_{13}P]^+$	1124.4501	1124.4518	1.5
MS/MS[a]	$[C_{49}H_{70}CoN_{12}O_7]^+$	997.4817	997.4837	2.0
MS/MS[a]	$[C_{46}H_{65}CoN_{10}O_6]^+$	912.4415	912.4435	2.2
MS/MS[b]	$[C_{46}H_{66}CoN_{10}O_6]^{2+}$	456.7244	456.7251	1.5
MS/MS[b]	$[C_{10}H_{18}N_2O_7P]^+$	309.0846	309.0850	1.3
MS/MS[b]	$[C_5H_9N_2]^+$	97.0760	97.0753	3.1

a: MS/MS of $[M+H]^+$ at CID 70; b: MS/MS of $[M+2H]^{2+}$ at CID 30.

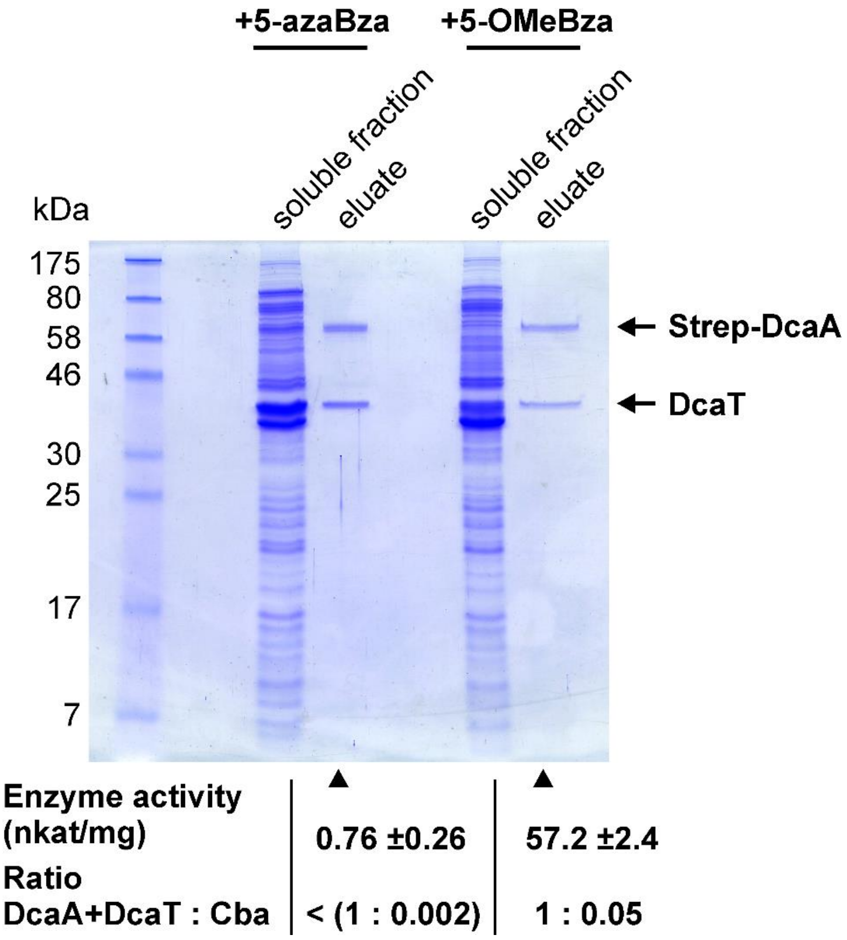

**Enzyme activity (nkat/mg)**	**0.76 ±0.26**	**57.2 ±2.4**
**Ratio DcaA+DcaT : Cba**	**< (1 : 0.002)**	**1 : 0.05**

Figure S14: Purification of recombinant Strep-DcaA. The soluble fractions (10 µg protein) and the eluates (1 µg protein) were separated on a 12.5% SDS/PAGE (Coomassie-stained).
